# Evaluating AI performance on end-to-end data science projects

**DOI:** 10.1371/journal.pone.0361887

**Published:** 2026-11-11

**Authors:** Evelyn Hughes, Rohan Alexander

**Affiliations:** 1 Department of Mathematics, University of Toronto, Toronto, Ontario, Canada; 2 Faculty of Information and Department of Statistical Sciences, University of Toronto, Toronto, Ontario, Canada; PLOS, UNITED KINGDOM OF GREAT BRITAIN AND NORTHERN IRELAND

## Abstract

Generative AI is used for knowledge work but many current AI evaluations focus on isolated tasks and constituent parts such as writing code, rather than end-to-end workflows. We test whether AI models can generate end-to-end data science projects. This requires AI models to select a dataset, develop a research question, write code to download, clean, and test data, create figures, and write a reproducible paper. To do this we collect 40 real end-to-end data science projects, then grade them according to a rubric. We use these to build an automated grading pipeline that evaluates data science projects and aligns with the grades that human graders give. For each of nine AI models we then generate five projects and use the automated grader to evaluate them. We find the extent to which generative AI models can complete end-to-end data science projects varies considerably by model. Most recent models did well on structured tasks, but there were considerable differences on tasks that needed judgment such as explaining measurement, writing abstracts, and citing sources. Anthropic’s Claude Opus 4.8 averaged 87 per cent and OpenAI’s GPT-5.4 averaged 85 per cent, both around the level of an A for an undergraduate. No model was consistently the best across all aspects of the rubric. These findings suggest that while AI models could soon approximate entry-level data scientists on routine tasks, they require consistent verification and evaluation.

## 1 Introduction

Data science is “the process of generating insight from data through reproducible and auditable processes” [1]. There is much more to data science than just writing code to build models. It requires blending a variety of skills including mathematical, computational, and statistical foundations; data management, curation, visualization, modeling and assessment; workflow and reproducibility; communication and teamwork; domain-specific knowledge; and ethics [2]. There is a difference between data science workflows in theory and in practice [3]. Each skill involves both technical and non-technical decisions, any of which could affect the overall outcome. Data science requires putting these skills together in a workflow that shows high-quality judgment. This broader focus makes evaluation focused not just on constituent skills, but also on the broader reproducible workflow, important.

There is considerable interest in the effect of generative AI on knowledge work, of which data science is an instance. AI has been found to intensify work by expanding task scope and accelerating pace [4] and to increase productivity in an experimental setting [5]. AI is affecting both the quality and quantity of scientific work [6,7]. DataSciBench is a benchmark of data science tasks that includes cleaning, exploration, visualization, and predictive modeling and uses predefined prompts with automated metrics to evaluate performance [8]. And research has looked at how participants could use tools such as ChatGPT to conduct data analysis [9]. But this previous work may not capture the judgment required in real-world analysis where data scientists must formulate questions and make decisions without explicit instructions. Similarly, previous work focusing on code generation for data [10,11], evaluating AI performance in machine learning, econometrics, or data analysis [12–14], or looking at specific data analysis tasks [15] does not necessarily consider the workflow and communication aspects that are critical for data science and many other knowledge work jobs.

Understanding where AI succeeds and fails on integrated workflows is important to understand its potential economic impacts. We evaluate generative AI on end-to-end data science projects. Our evaluation tests the extent to which generative AI models can develop questions, code in Python, clean and prepare data, create visualizations, and write a paper. This is closely related to the work of professional data scientists [16].

We collect 40 projects that university undergraduates initially wrote. Two human experts evaluated each project on a 17-category rubric totaling 45 points. The A-/B+ threshold for undergraduates on these projects is 80 per cent. We use this collection to develop an automated grading pipeline using LLM-as-a-judge. We then use that to evaluate five projects from each of nine AI models.

While it is important to not over-index on the specific numbers, overall, on average, Anthropic’s Claude Opus 4.8 got 87 per cent; Claude Opus 4.6 and OpenAI’s GPT-5.4 got 85 per cent; GPT-5.2 got 82 per cent; Google’s Gemini 3 Flash got 78 per cent; xAI’s Grok-4 got 70 per cent; Gemini 3 Pro got 59 per cent; Meta’s Llama 4 got 51 per cent; and OpenAI’s older GPT-4o model got 32 per cent, while GPT-5.5 was unable to complete the task. Scores varied between the different rubric categories. Models tended to do better on structured tasks, such as writing titles and test suites. There was larger variance on tasks that needed judgment, such as writing about measurement, writing an abstract, and including correct citations.

The contribution of our paper is to evaluate generative AI models on end-to-end data science projects. We find that most recent models produce work that is similar to a good undergraduate. They do well on tasks that are well-defined, but less well on tasks requiring judgment, such as discussing measurement or writing abstracts. And there are considerable differences between models, even from the same model provider.

## 2 Materials and methods

We want to systematically evaluate the extent to which AI can complete data science projects. To do this we first collect human-written and evaluated projects, then we use those to develop and validate an auto-grader. We then prompt models to generate projects. Finally, we use the auto-grader to evaluate those in a scalable way.

Our workflow, which we developed for this paper, is summarized in Fig 1.

**Fig 1:**
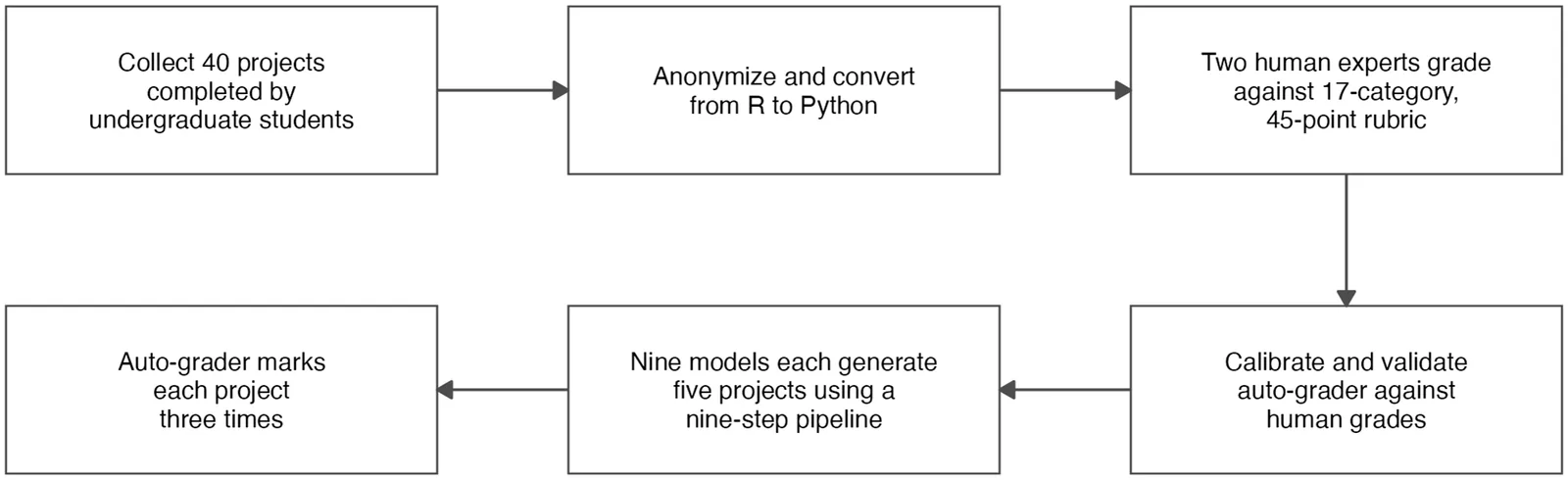
Overview of our evaluation process. We collect and standardize 40 student-completed projects, which two human experts grade against a 17-category rubric. These human-graded projects are used to calibrate and validate an LLM-based auto-grader, which then grades the projects generated by each AI model.

The projects involve the full data science lifecycle: acquisition, cleaning, validation/testing, visualization, communication. The task is of moderate difficulty, and can usually be completed well by upper-year undergraduates. The setting is primarily academic, although it uses real data from the City of Toronto.

### 2.1 Evaluation

Benchmarking is one way to evaluate model capabilities. A benchmark brings together a dataset of tasks and a performance metric [17]. There are legitimate criticisms of being solely focused on benchmark performance [18,19], especially concern about selective disclosure [20]. Nonetheless, evaluation against a benchmark, particularly over time, is a widely used way to track AI progress.

OpenAI publicly released GPT-3 in 2020, catalyzing considerable focus on publicly sharing benchmarks around AI code-generation. For instance, HumanEval [21] evaluated the capacity of models to write Python functions from docstrings. Similarly, MBPP [22] was a larger benchmark consisting mostly of Python programming problems. More recently, SWE-bench [23] measures AI progress on writing Python code to resolve real GitHub issues. These, and similar, benchmarks have various degrees of complexity and real-world relevance, but all evaluate the capacity of models to generate code within a particular setting, rather than measuring capacity to complete a project end-to-end.

To construct our evaluation we collect 40 projects initially completed by university students and anonymize them. Two human experts then use a 17-category rubric to mark each project and resolve any differences.

The task is to complete a data analysis project using data from Open Data Toronto. We adapted this task from an upper-year undergraduate assignment at the University of Toronto. Students can pick any dataset they want from Open Data Toronto. Students work individually and submit a link to a repository that includes code and a short paper telling a story about the data. The entire work needs to be fully reproducible. Students are provided with an example GitHub repo and this provides more information about the steps they should take and what should go into the paper. In addition to the detailed instructions, students also receive the rubric used to mark their submission and they can see past examples.

The rubric was developed for students following a specific workflow [24]. And it was refined over multiple years of teaching. The different groups of the rubric map into the different data science pipeline stages and represent many of the key skills required to do data science. It was refined for this paper during the process of the two authors grading the example human submissions.

Each project must include a Python script, 01-download.py, that downloads a dataset from Open Data Toronto. A second script, 02-clean.py, cleans and prepares the data. Students then test the analysis dataset, using 03-test.py, to check there are no obvious issues. They usually do this using the Python pydantic package. Students write the paper as a Quarto document, paper.qmd, which compiles to a PDF, paper.pdf. The Quarto document integrates the code required to generate graphs and tables with text. The required sections of the paper are: Introduction, Data, Results, and Discussion. Students must cross-reference all graphs and tables. The paper also needs to include citations that compile using a BibTeX file, references.bib. The paper can be considered a technical report and is the main communication deliverable. Finally, students must ensure reproducibility by including a UV lock file and pyproject.toml or requirements.txt, a detailed README.md, and a well-structured repository.

We obtained project repositories from 40 students by asking them, individually, if they were willing to let us use their repository for this purpose. We did this after the class ended and we finalized and released grades. Students could complete their projects using either R or Python. To make our evaluation process easier and more consistent, we converted projects that originally used R to Python by making the minimal number of changes needed. This involved changing the packages and main functions such as from dplyr to pandas, or from ggplot2 to matplotlib. We anonymized the projects by changing the name and emails.

For each project two human graders independently evaluated it against a detailed rubric. The rubric has 17 categories, totaling 45 points, and we combine them into six groups: Reproducibility, Presentation, Analysis, Graphs, Referencing, and Writing (Table 1). Each category has points associated with it and detailed instructions about what is required for each level. When the markers disagreed they resolved differences through discussion, and we added additional detail to the rubric instructions. The full rubric is available in S1 Appendix.

**Table 1:** Grading rubric for end-to-end data analysis projects. The rubric has 17 categories grouped into six evaluation types, totaling 45 points: reproducibility (9 points); presentation (10 points); analysis (10 points); graphs (8 points); referencing (4 points); writing (4 points).

Group	Category	Max points
Reproducibility	Reproducible workflow	5
	Tests	3
	Author, date, and repo	1
Presentation	Title	2
	Abstract	4
	Introduction	4
Analysis	Measurement	2
	Data	4
	Discussion	4
Graphs	Data tables	2
	Results figures	4
	Captions	2
Referencing	Cross-references	1
	References	2
	Python cited	1
Writing	Professionalism	3
	Banned words	1

### 2.2 Auto-grader development and validation

Assessing the projects created by the generative AI models in a consistent, scalable way required developing and validating an auto-grader.

By way of background, the question of how to evaluate responses to the tasks in a benchmark against the desired response is a common problem with benchmark evaluation. For open-ended tasks it can be difficult to uniquely determine what the answer should be and so researchers commonly define rubrics. There are two ways to evaluate submissions against a rubric: human-evaluation or LLM-as-a-judge. The latter has the benefit of being scalable, and with appropriate set-up can approximate human preferences [25]. That said, there are systematic biases from LLM-as-a-judge, including position and verbosity bias [26] and particular concerns when specialized knowledge is required [27].

We use an automated approach because it allows us to evaluate many more submissions from the generative AI models than would be viable using human evaluation. It also allows us to evaluate new models as they are made available. We use LLM-as-a-judge with per-category evaluation.

We use our benchmark to calibrate the auto-grader. We first had the auto-grader, using GPT-4o as a judge, mark ten papers in the benchmark set and compared the marks and comments generated by the auto-grader with the marks and comments provided by the humans in the benchmark. We also checked for consistency across multiple markings of one paper. Where there were differences we changed the prompts and rubric categories to improve clarity. We also made more substantial changes such as grading each rubric category in its own API call and providing each aspect of the submission separately. The final evaluation was done on all projects. After finalizing this set-up we evaluated both GPT-5.2 and Claude Sonnet 4.5 as a judge, with each model grading all projects three times. The mean absolute difference from the human total mark for Claude Sonnet 4.5 was 3 points out of 45 and was 6.3 points out of 45 for GPT-5.2 (Fig 2). Even though they differed on the exact mark and Sonnet 4.5 was higher than GPT-5.2, the two models produced correlated rankings (r = 0.85). Both graders were internally consistent when presented with the same project, with mean within-project standard deviations below 1.0 point. Because Sonnet 4.5 was closer to the human mark we used Sonnet 4.5 for the auto-grader. This produced an automated grading pipeline that provides an average overall mark within 10 per cent of the human-provided total mark, and is not substantially different on any particular category.

**Fig 2:**
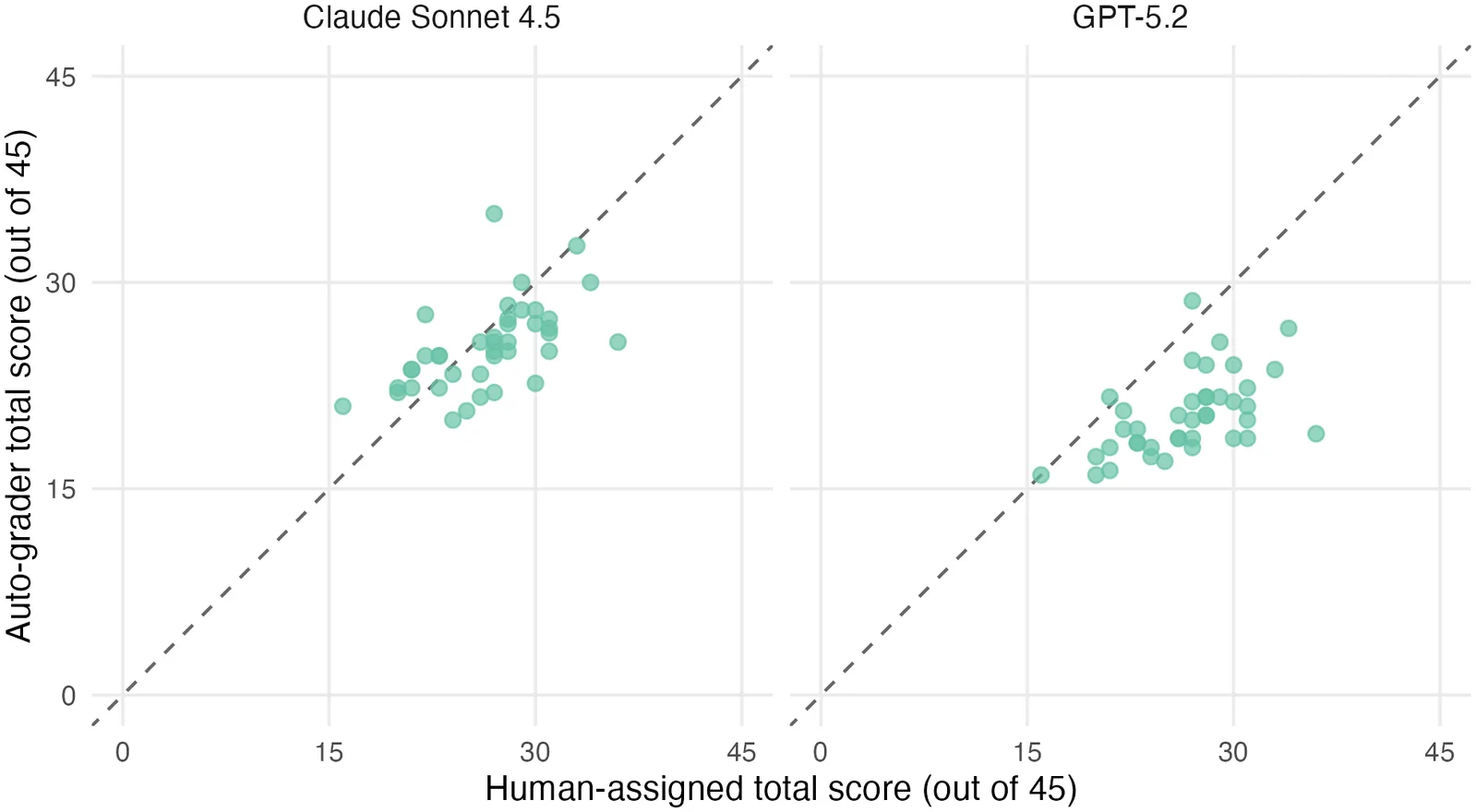
Comparison of human-assigned and auto-grader total scores for the 40 benchmark projects, for each potential judge model. Each point is one project; the auto-grader score is the mean across three independent grading runs. The dashed line is perfect agreement between the human score and the model score. Claude Sonnet 4.5 is closer to human grading (mean absolute difference 3.0 points out of 45) than GPT-5.2 (6.3 points).

In its final form, our automated grading system is underpinned by Claude Sonnet 4.5 only. Each category is a separate API call. We prompt the model with the category, the evaluation criteria, the score options and explicit descriptions, and the relevant project material. For reproducibility, the model receives all scripts, the README, and project management files. For writing, it receives paper.qmd. For figures it receives both the source code and the rendered PDF as images. The model returns category name, numeric score, and a brief comment, as semicolon-separated values. The system prompt specifies the need for publication-level standards, to avoid being influenced by jargon or verbose writing, and that the model should return a lower mark if it is not sure between two options. Table 2 specifies the inputs provided to the grading model for each evaluation type.

**Table 2:** Input materials provided to the LLM grading model for each evaluation type. Each category is evaluated individually to reduce context and position bias. Reproducibility evaluation requires the complete codebase; Presentation and Analysis require only the manuscript source; Graphs evaluation includes rendered PDF images to assess visual quality.

Group	Input materials	File types
Reproducibility	Scripts, README, requirements.txt	.py, .txt, .md
Presentation	Paper source	.qmd
Analysis	Paper source	.qmd
Graphs	Figure source, rendered output	.qmd, .pdf (as images)
Referencing	Paper source, bibliography	.qmd, .bib
Writing	Paper source	.qmd

### 2.3 Project generation pipeline

We can now have the AI models generate projects. This involves prompting each model to generate projects using a nine-step pipeline (Table 3). Each step is a separate API call. We do this because we often found the models got stuck on some component that prevented them from being able to return a complete project. The history maintains context, but this does mean that we are erring on the side of providing the models with every opportunity to succeed in the task. A more strict evaluation would remove this separation. The models receive the relevant portion of the rubric for each step. The full rubric is in S1 Appendix.

**Table 3:** Nine-step pipeline for LLM-generated data analysis projects. Each step is a separate API call with conversation history maintained across steps. The pipeline progresses from dataset selection through code generation, quality assurance, and final documentation.

Phase	Step	Output
Data acquisition	1. Planning	Dataset selection, research question
	2. Download	01-download.py
	3. Clean	02-clean.py
Quality assurance	4. Testing	03-test.py (pytest suite)
	5. Requirements	requirements.txt or uv files
	6. References	references.bib
Documentation	7. Figures	figures.qmd (visualizations)
	8. Paper	paper.qmd (final manuscript)
	9. README	README.md (reproduction guide)

The first phase is data acquisition and comprises three steps: planning, data download, and data cleaning. In Step 1 (planning), we provide the model with three datasets from Open Data Toronto from which it can pick: light duty city vehicle utilization data, deaths of people experiencing homelessness, and neighborhood profiles. This is more specific than what is provided to the students, because we found that the models often struggled to pick an appropriate dataset and this resulted in them being unable to do the rest of the process. We instruct the model to select one, develop a research question, and outline an analytical approach. In Step 2 (data download), we ask the model to write a Python script to download the selected dataset. This requires more than just pasting the dataset URL into a function because sometimes there are multiple datasets and the model must identify which one to use. The script saves data to data/raw/. In Step 3 (data cleaning), the model writes a cleaning script. To inform variable handling, the model receives the first two lines of each downloaded file. The script saves the cleaned and prepared dataset to data/clean/.

The second phase is quality assurance and reproducibility and involves two steps. In Step 4 (testing), the model creates a test suite, often using pydantic to verify data integrity, check columns, and data types, and validate value ranges. In Step 5 (requirements), the model is to deal with package management by generating a requirements.txt file.

The third phase involves four steps. In Step 6 (references), the model creates a BibTeX file with the papers that were cited, as well as citing the data source and software such as Python packages, as appropriate. In Step 7 (figures), the model creates a Quarto document (figures.qmd) generating visualizations. The pipeline renders this, and the model receives both source and output PDF. In Step 8 (paper), the model writes the final paper as a Quarto document, receiving the BibTeX file and rendered figures to include as needed. The paper must include title, abstract, introduction, data description, results, discussion, and references. In Step 9 (README), the model creates documentation explaining the research question, repository structure, and reproduction instructions. Generation costs are summarized in S2 Appendix.

## 3 Results

We evaluated nine AI models: Anthropic’s Claude Opus 4.8 and 4.6, Google’s Gemini 3 Flash and Gemini 3 Pro, OpenAI’s GPT-5.4, GPT-5.2 and GPT-4o, xAI’s Grok-4, and Meta’s Llama 4. Each model generated five projects. The auto-grader then graded each project three times against a rubric (available in S1 Appendix). We conducted analysis using R [28] and the tidyverse packages [29]. We also attempted to use OpenAI’s GPT-5.5 rather than GPT-5.4, but it exhausted our pipeline’s 100-retry cap without completing a project, while other models needed only between 5 and 24 attempts. So GPT-5.5 does not appear in our results.

### 3.1 Overall performance

When averaged across all five projects, Claude Opus 4.8 achieved the highest average score of 39 points (87 per cent), followed by Claude Opus 4.6, GPT-5.4, and GPT-5.2. Surprisingly, Gemini 3 Flash was also around the A-/B+ threshold, at 35 points (78 per cent), but Gemini 3 Pro at 27 points (59 per cent) was lower (Fig 3). In Fig 3 the dashed line at 36 points is the 80 per cent cut-off between an A- and a B + . Claude Opus 4.8 and 4.6, GPT-5.4 and GPT-5.2, and Gemini 3 Flash are all around this level of performance. Grok-4, Gemini 3 Pro, Llama 4, and the older GPT-4o model, are below it. Examples of the first pages of the papers produced by different models are in S3 Appendix.

**Fig 3:**
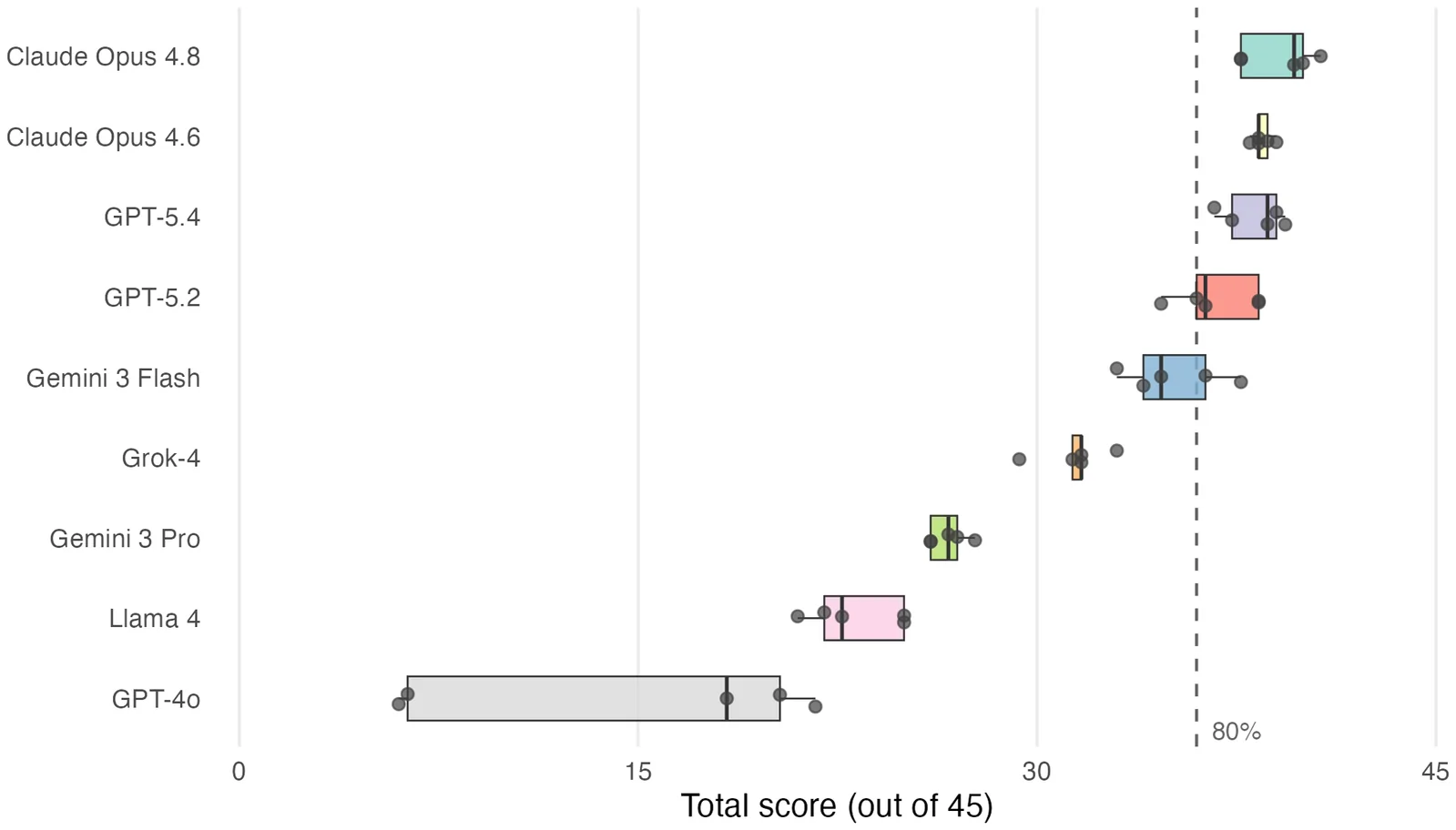
Overall performance of nine AI models on an end-to-end data analysis project. The maximum score is 45, and the dashed line at 36 (80 per cent) is the threshold between a B+ and A-, which is the typical performance of good upper-year undergraduates.

### 3.2 Performance by category type

Fig 4 shows scores aggregated by evaluation type. Anthropic’s Claude Opus 4.8 and 4.6 did well across most types, as did OpenAI’s GPT-5.4 and GPT-5.2.

**Fig 4:**
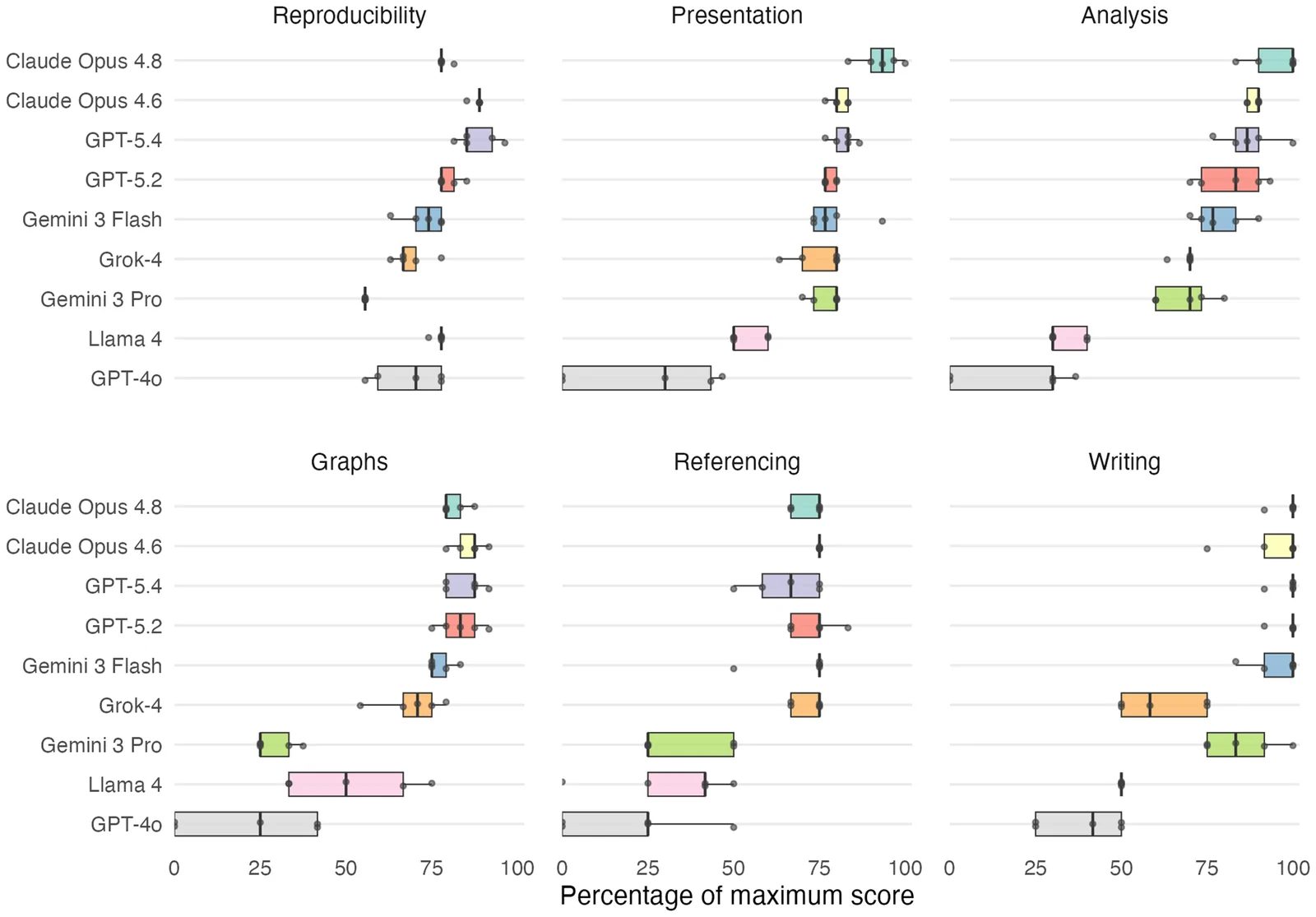
Model performance by rubric evaluation type shown as the percentage of maximum possible points for that type. Evaluation types and maximum number of points are: Reproducibility (9 points), Presentation (10 points), Analysis (10 points), Graphs (8 points), Referencing (4 points), and Writing (4 points).

Reproducibility (9 points) considers code quality and documentation. Good projects tended to have extensive test suites and detailed READMEs. Worse scores tended to have worse documentation. Claude Opus 4.6 and GPT-5.4 led on this, with both averaging 88 per cent. Writing (4 points) showed less difference between models, but this was partly due to the difficulty of automatically evaluated writing. For instance, one key part was avoiding a specific list of words, and some models were unable to do this. This tests direct instruction-following because the rubric included the list of words to avoid.

Although Claude Opus 4.8 achieved the highest total score, no single model led on all six evaluation types. Claude Opus 4.8 was strongest on Presentation (93 per cent) and Analysis (95 per cent), but not on Reproducibility (79 per cent), Graphs (82 per cent) or Referencing. The best-performing model depends on which aspects of the data science workflow are weighted most heavily.

### 3.3 Performance by rubric task

When looking at the detailed average scores by rubric item there are clear areas of strength and weakness (Fig 5). Many models achieved perfect or near-perfect scores on Title (2/2). But more extensive writing requirements, such as the Introduction and Discussion resulted in differences between models. For instance, Claude Opus 4.6 scored 3.27/4 on Introduction, while Llama 4 scored 2/4. Another writing aspect of interest is writing an abstract (4 points). This is especially interesting because an abstract is so short yet must communicate so much detail. No model averaged full marks across its five projects. The best was Claude Opus 4.8 at 3.5/4, while Llama 4 scored 2/4. This suggests that even just evaluations focused on automated abstract evaluation could be useful. Writing about data was another area where there were considerable differences between models. For instance, Claude Opus 4.6 averaged 3.2/4 and GPT-5.2 averaged 3.1/4, while Gemini 3 Pro averaged 2.3/4. Lower-scoring projects tended to not discuss the reasons for variable selection or cleaning decisions.

**Fig 5:**
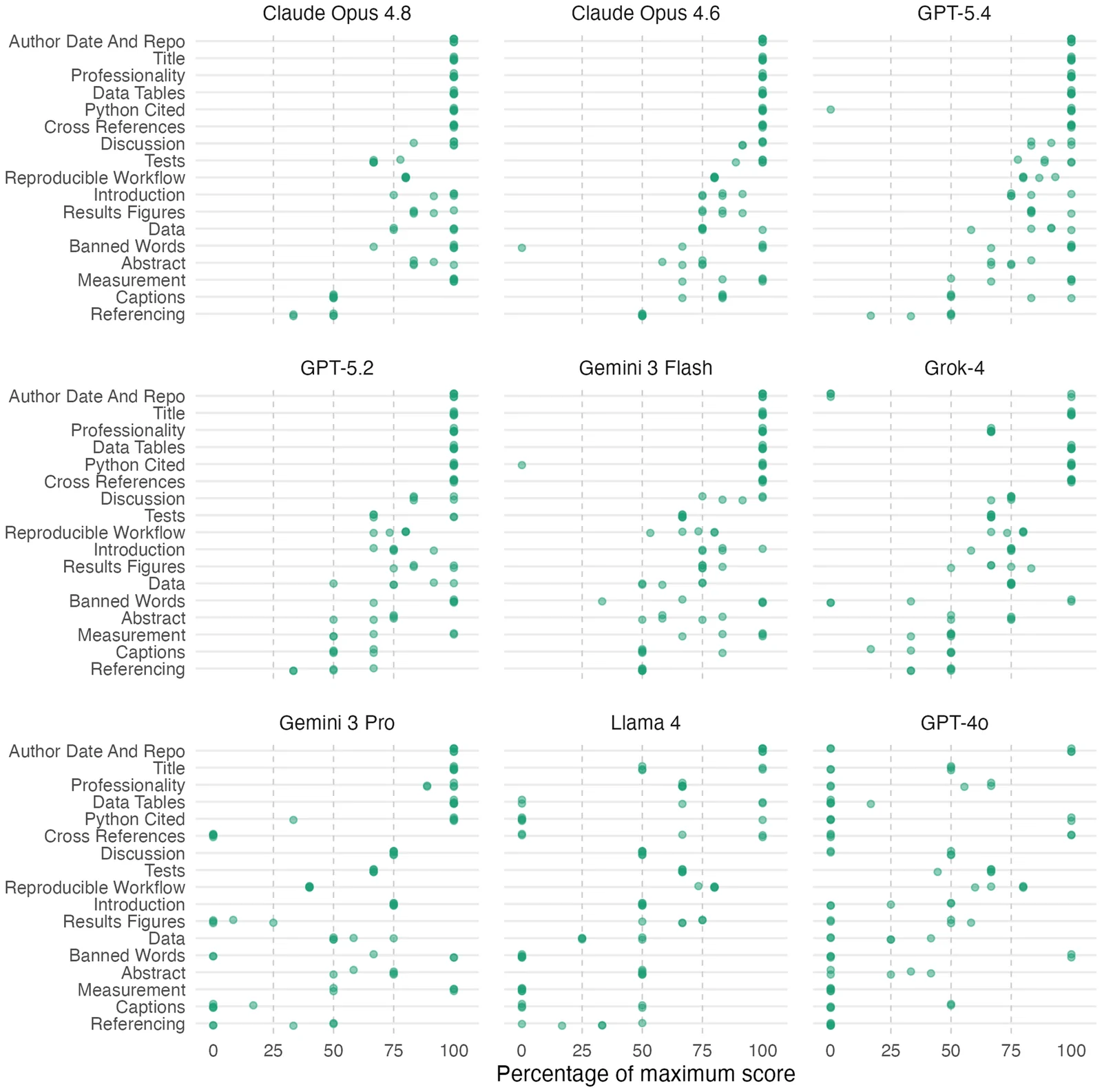
Model performance for all 17 rubric categories shown as percentage of maximum possible points for that category. Categories are ordered by average performance across all models (highest at top). Each point is one of the model’s five projects.

It was surprising that not every model got full marks for developing a test suite (3 points). This required consideration of potential failure modes for the data and writing tests for them. Claude Opus 4.6 achieved an average of 2.93/3. Surprisingly, Claude Opus 4.8, which tended to be the best model overall, scored only 2.07/3 on tests. Strong test suites considered data types, value ranges, and null handling, while weak ones tended to be overly generic.

The measurement category required explaining how some real-world phenomenon becomes a dataset entry. The ability to think deeply about this is important for data scientists and the lower performance of many models on this may reflect a failure to put a given dataset in context. It was also surprising that Gemini 3 Pro received zero marks for cross-references because cross-referencing is well-documented and uses code.

The models generally did well on template-following tasks such as writing a title or putting together a test suite. Tasks that involved synthesis and context, such as writing an introduction or discussion section, or writing code to make figures, differentiated models. And data science specific tasks such as measurement, cross-references and citations received consistently lower marks, with only a couple of exceptions. A common referencing failure was generating malformed BibTeX entries for Python packages, which produced placeholder citations such as “matplotlib2023?” with question marks instead of publication details.

### 3.4 Auto-grader consistency

Claude Sonnet 4.5 graded each of the 45 model-generated projects three times. Table 4 shows the standard deviation for each grading run, for each model’s five projects, as well as the average. The overall average standard deviation was 0.8 points out of 45.

**Table 4:** Grading consistency across three evaluation runs per project. Each cell shows the standard deviation of total scores across three independent grading runs for that project. Low values indicate the auto-grader assigns similar scores when re-evaluating the same project.

Model	Project 1	Project 2	Project 3	Project 4	Project 5	Mean
Claude Opus 4.8	0.6	1.2	0.6	0.0	0.6	0.6
Claude Opus 4.6	0.6	1.0	1.5	1.5	0.0	0.9
Gemini 3 Flash	0.0	0.6	1.2	0.0	0.6	0.5
Gemini 3 Pro	0.0	0.0	0.6	1.5	0.0	0.4
GPT-4o	1.2	0.6	1.2	0.0	0.6	0.7
GPT-5.2	0.6	0.6	1.7	1.2	0.6	0.9
GPT-5.4	0.6	1.5	1.2	1.7	1.2	1.2
Grok-4	2.1	1.0	1.5	0.6	0.6	1.2
Llama 4	1.2	1.0	1.0	0.0	0.0	0.6

## 4 Discussion

There is considerable interest in the extent to which AI will replace white-collar work. Our findings suggest even the best current AI models still require human oversight. Claude Opus 4.8 and 4.6, and OpenAI’s GPT-5.4 and 5.2 all did well, but still had notable weaknesses, while GPT-5.5 could not complete the task. Surprisingly, Gemini 3 Pro scored lower than Gemini 3 Flash. Gemini 3 Pro required more retry attempts during project generation, which may have decreased output quality. Broadly, comparing newer models with older ones suggests that continued development, or data-science-specific models, could further increase performance. This also points to the need for more domain-specific benchmarks as we examine AI performance on specific areas of work. At the moment, an organization interested in deploying AI for data science cannot simply assume that just any model could be used for the task without considerable evaluation and verification.

Models tended to do well when they could follow well-defined templates or patterns such as Title, and Author/Date/Repo, and do worse in sections such as Abstract, Introduction, and Discussion. Papers written by models tended to do worse in these sections not because they were poorly written—they were not—but because that writing tended to not communicate much information. Despite the presence of the “right words”, they did not seem to communicate deep expertise and thoughtful reflection. However it was challenging to automatically evaluate writing at any deep level and again, task specific benchmarks may help improve this.

Measurement required describing how some phenomena become an entry in a dataset. The difficulties that many models had on this may reflect it being underrepresented in training corpora despite being critical in data science. Similarly, lower marks on cross-references suggests a failure to understand Quarto syntax even though the instructions referenced material with explicit examples. While difficulties with a specialized tool like Quarto may not be specifically concerning, it may telegraph difficulties when translating performance on general benchmarks into delivering return on investment for businesses.

The best models, Claude Opus 4.8 and 4.6, GPT-5.4 and GPT-5.2, and Gemini 3 Flash, scored around the same as a good undergraduate. Models may soon be able to complete routine data analysis with minimal revision, but the systematic weaknesses we document would mean users still need to check them. Fully autonomous deployment for data science may carry substantial risk without extensive evaluation.

We did not prompt engineer our models for dataset-specific analysis. Better performance would be possible with this, but this may undermine real-world value. Data scientists try to learn from data, conducting different analyses on different datasets. That said, with further prompt engineering we may be able to remove some of the guardrails we needed, such as breaking down the steps into separate API calls and providing the model with a choice from a handful of datasets. The evaluation of visualization is difficult and taste is difficult to evaluate. The high variance we find in figure-related categories may partly reflect evaluation limitations.

LLM-as-a-judge introduces a variety of biases [26]. Of particular concern is whether a grading model favors output from its own provider. Our grader is Anthropic’s Claude Sonnet 4.5, and Anthropic’s Claude Opus 4.8 and 4.6 are the two highest-scoring models. It is worth noting that both Claude Sonnet 4.5 and GPT-5.2 graded the 40 human-written benchmark projects and they tended to agree on the order of the projects, although they disagreed on the actual mark. And the difference in the marks given in repeated gradings of the same project is small. Broadly the Anthropic-based grader scored the best OpenAI submissions essentially the same as the best Anthropic submissions. Cross-grading all model outputs with a second judge from a different provider would be important in future research designs. A related concern is that while our auto-grader calibration used only 10 of the 40 projects and used a different judge (GPT-4o) than the final grader (Sonnet 4.5), we conducted validation using all 40, so no strictly held-out human set exists.

Even if Claude Opus 4.8 were performing, say, only on par with recent models from Google and OpenAI, that level of performance would still be comparable to that of a good undergraduate. While our rubric emphasizes academic aspects that businesses may be less concerned with, citation checking is a relatively easy way to detect hallucinations. That said, producing an academic-style paper is not a standard component of industry data science pipelines and future work should examine AI performance on other forms of communication, such as reports or dashboards. We also focus on evaluating analysis conducted on data from the City of Toronto, and findings may be different in other domains.

Our sample is small and this meant we did not use statistical methods to look for differences between models. Our finding that, say, some model was the best, and that some other model was second, should be more broadly considered as a grouping and indicative of the general performance level of recent models, rather than a definitive ranking. It is also surprising that GPT-5.5 was unable, repeatedly, to complete the task.

This paper evaluates something many existing evaluations do not, which is whether an AI model can complete a data science project end-to-end. This involves choosing a question, cleaning data, testing data, developing visualizations and communicating findings in a reproducible paper. We calibrate an automated grader based on the grades of projects completed by undergraduates. Our evaluation matters because data scientists are typically employed to deliver integrated workflows. Future work should continue to increase the number of projects per model and reduce the guardrails our paper needed. It would also be interesting to consider how models could perform when used alongside human guidance.

## Supporting information

S1 Appendix Complete grading rubric. The rubric has 17 categories grouped into six evaluation types, totaling 45 points, with criteria for each score level.
(PDF)

S2 Appendix Generation costs. Cost, time, and retry attempts for generating five projects per model, and the cost of the GPT-5.5 attempt.
(PDF)

S3 Appendix Example generated papers. First pages of six generated papers illustrating the range of output quality.
(PDF)
