# Supplementary material for: Evaluating AI performance on end-to-end data science projects
Source: PLoS One. 2026 Nov 11;21(11):e0361887. doi: 10.1371/journal.pone.0361887 (PMC13754138; doi:10.1371/journal.pone.0361887)
Supplement: Supplementary file 1 [file pone.0361887.s001.pdf]

S1 Appendix. Rubric details

The rubric has 17 categories grouped into six evaluation types, each with explicit criteria per score level (Table 1). For example, Measurement (2 points) awards 2 for "thorough and specific" explanation of how phenomena become data entries, 1 for "some explanation but lacks depth," and 0 for "little or no explanation."

Table 1. Complete grading rubric for end-to-end data analysis projects. The rubric has 17 categories grouped into six evaluation types (Reproducibility, Presentation, Analysis, Graphs, Referencing, Writing), totaling 45 points. Each category includes specific evaluation criteria and scoring descriptions for each point level.

Type	Category	Points	Key Criteria	Scoring
Reproducibility	Reproducible Workflow	5	Code is thoroughly documented code, including preamble, comments, nice structure, and is styled. Hard-coded paths are avoided. A detailed README.md is included as well as a project libraries management file, such as uv.lock or requirements.txt. No more than 5 scripts to run	0 – 'Disorganized; lacks comments; not reproducible' 1 – 'Partial structure; some documentation; hard to reproduce' 2 – 'Missing two or more major components; still somewhat reproducible' 3 – 'Missing one major component; still somewhat reproducible' 4 – 'Clear structure and documentation; readable and reproducible with minor friction' 5 – 'Fully reproducible; all components present'
Reproducibility	Tests	3	Test suite exists in its own script Tests go beyond basic null/invalid checks Tests use proper framework (unittest or pytest) Test suite is extensive and sophisticated	0 – 'No or few tests' 1 – 'Basic or minimal tests written' 2 – 'Organized suite covering some major components with structure' 3 – 'Extensive and sophisticated test suite using frameworks like pytest or unittest'
Reproducibility	Author, Date, & Repo	1	Author name is clearly included Date of submission in unambiguous format is included Link to GitHub repo is included	0 – 'One or more elements missing or unclear' 1 – 'All three (author, date, GitHub repo) are clearly included'

(continued)

Type	Category	Points	Key Criteria	Scoring
Presentation	Title	2	Title is informative (not generic like ”Paper X”) Title explains the story Subtitle conveys the main finding No puns used	0 – ‘Missing or generic’ 1 – ‘Informative but not fully representative of paper’ 2 – ‘Clear, specific, and polished. Includes subtitle with key finding’
Presentation	Abstract	4	Pitched to non-specialist audience Explains what was done Explains what was found Explains why this matters Includes scene setting States the research question Makes clear what we learn about the world Specific about findings (includes ”when” and ”how much”) Not overly brief	0 – ‘Missing or lacks two or more required elements’ 1 – ‘Most elements present but unclear or inaccessible to general audience’ 2 – ‘Clear and complete but too wordy, or technical’ 3 – ‘Clear, concise (~4 sentences), pitched to general audience’ 4 – ‘All elements covered in clear, concise prose with relevance highlighted’
Presentation	Introduction	4	Provides broader context to motivate Details what the paper is about Identifies a clear gap that needs to be filled States what was done States what was found (specific results) Explains why it is important Outlines the structure of the paper Self-contained (reader can understand without rest of paper) Appropriate length (3-4 paragraphs or ~10% of total) Thorough, not overly brief	0 – ‘Missing or incomplete’ 1 – ‘Some elements included, but lacks clarity or structure’ 2 – ‘Most elements present, some issues with clarity’ 3 – ‘Clear and logically structured’ 4 – ‘Fully self-contained; explains motivation, findings, and structure’
Analysis	Measurement	2	Explains how real-world phenomena becomes data entry Describes collection method Describes measurement method Describes categorization/coding method Specific and thorough explanation	0 – ‘Vague or missing’ 1 – ‘Some explanation’ 2 – ‘Thorough and specific’

(continued)

Type	Category	Points	Key Criteria	Scoring
Analysis	Data	4	Discusses alternative datasets and why not used Mentions if variables were constructed Notes high-level cleaning aspects Focuses on destination, not journey Walks reader through variables of interest Not doing EDA in this section Uses appendices if too detailed	0 – 'Missing or incoherent' 1 – 'Described but with major omissions' 2 – 'Adequate with minor clarity or rationale issues' 3 – 'All variables of interest explained; some polish missing' 4 – 'Thorough and clearly justified; All variables of interest are clearly explained'
Analysis	Discussion	4	2-3 sections (one per key takeaway) Each takeaway clearly highlighted & explained in detail Explains what results mean relative to research question Connects findings back to introduction motivation Acknowledges uncertainties and avoids overclaiming Goes beyond restating numbers and explores real-world implications Discusses potential confounding factors	0 – 'Results restated without meaning' 1 - "Unclear interpretation' 2 – 'Interpretation present but shallow' 3 – 'Results explained in context with some weak points' 4 – 'Strong, thoughtful interpretation linked to findings and limitations'
Graphs	Data Tables	2	Graphs and/or tables showing what data looks like Summary statistics table is included Table contains data.describe() or equivalent Located in the DATA SECTION	0 – 'Missing or insufficient' 1 – 'Table is present in the DATA SECTION, but it does not contain summary statistics.' 2 – 'There is a table in the DATA SECTION containing summary statistics.'
Graphs	Results Figures	4	Analytically focused graphs/tables present Journal-quality appearance Clear and digestible Serves clear purpose and supports claims Fully self-contained Properly labeled (NO variable names as labels) For tables: no excessive decimal places No boxplots (or if present, actual data overlaid)	0 – 'Missing or unreadable' 1 – 'One or more present but poorly formatted or unclear' 2 – 'Clear and interpretable' 3 – 'Well-designed and polished' 4 – 'Clear, polished, labeled, and directly supports claims'

(continued)

Type	Category	Points	Key Criteria	Scoring
Graphs	Captions	2	All figures and tables have captions Captions are detailed and meaningful Main point clear without reading text Does NOT simply state figure type (e.g., "Histogram of...")	0 – 'Most missing or meaningless' 1 – 'Present but not sufficiently detailed' 2 – 'All figures/tables have meaningful, standalone captions'
Referencing	Cross References	1	All figures and tables are labeled All figures and tables referenced in text (e.g., @fig-x)	0 – 'No cross-references or missing cross-references' 1 – 'All visuals and equations numbered and referred to in the text'
Referencing	Referencing	2	All citations correctly capitalized and formatted No reversed names (e.g., "Data, Toronto Open" should be "Open Data Toronto")	0 – 'Poor or missing citations' 1 – 'Citations have minor formatting issues' 2 – 'Complete and correctly formatted citations'
Referencing	Python Cited	1	Python given in text citation Python appears in reference list Python packages cited appropriately	0 – 'Python is not cited' 1 – 'Python is cited'
Writing	Professionality	3	Free of noticeable typos Grammatically correct Coherent, concise and clear Mature tone, not overly technical or wordy	0 – 'Numerous grammatical/spelling issues' 1 – 'Readable but with issues in clarity or flow' 2 – 'Clear and mostly error-free' 3 – 'Polished and mature'
Writing	Banned Words	1	check if the text contains any of the following words: advanced, comprehensive, critical, crucial, data-driven, delve/s, imperative, insight/s	0 - 'One or more of the words are used on the list' 1- 'None of the words on the list are used'
