# Supplementary material for: Evaluating AI performance on end-to-end data science projects
Source: PLoS One. 2026 Nov 11;21(11):e0361887. doi: 10.1371/journal.pone.0361887 (PMC13754138; doi:10.1371/journal.pone.0361887)
Supplement: Supplementary file 2 [file pone.0361887.s002.pdf]

S2 Appendix. Generation costs

Table 1 summarizes the computational cost of generating the five projects for each model. Total cost includes all API calls across the nine-step pipeline.

Retry attempts occur when a step fails (e.g. a script produces an error) and the pipeline re-prompts the model with the traceback. Apart from GPT-5.5, Llama 4 and Gemini 3 Pro required the most retries (24 and 22), which is consistent with their lower scores. We provided GPT-5.5 with 100 retry attempts, which cost $5.61 and took 43 minutes, yet it completed no projects.

Grading the model-generated projects three times each using Claude Sonnet 4.5 took around four hours. And validating the auto-grader using GPT-5.2 took around three hours.

Table 1. Cost and time to generate five projects per model. Total cost reflects all API calls across the nine-step generation pipeline. Retry attempts indicate how many pipeline steps required re-prompting after failure. Models requiring more retries tended to score lower.

Model	Time (min)	Cost (USD)	Retries
Claude Opus 4.8	20	$6.18	5
Claude Opus 4.6	42	$4.75	5
Gemini 3 Pro	73	$3.31	22
GPT-5.4	16	$2.58	5
Grok-4	47	$1.73	8
GPT-5.2	34	$1.73	5
GPT-4o	17	$1.06	6
Gemini 3 Flash	26	$0.39	7
Llama 4	79	$0.32	24
