# Supplementary material for: Evaluating AI performance on end-to-end data science projects
Source: PLoS One. 2026 Nov 11;21(11):e0361887. doi: 10.1371/journal.pone.0361887 (PMC13754138; doi:10.1371/journal.pone.0361887)
Supplement: Supplementary file 3 [file pone.0361887.s003.pdf]

S3 Appendix. Example generated papers

To illustrate the range of output quality Fig 1 shows the first page of six generated papers. Higher-scoring papers have well-formatted titles, abstracts, and introductions. Lower-scoring papers have generic writing (Llama 4) and broken Quarto rendering (GPT-4o).

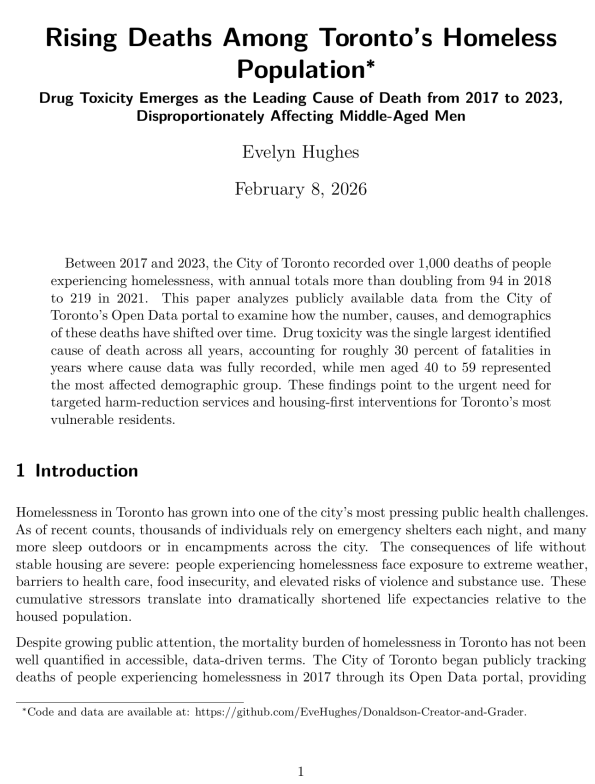

Rising Deaths Among Toronto's Homeless Population*

Drug Toxicity Emerges as the Leading Cause of Death from 2017 to 2023, Disproportionately Affecting Middle-Aged Men

Evelyn Hughes

February 8, 2026

Between 2017 and 2023, the City of Toronto recorded over 1,000 deaths of people experiencing homelessness, with annual totals more than doubling from 94 in 2018 to 219 in 2021. This paper analyzes publicly available data from the City of Toronto's Open Data portal to examine how the number, causes, and demographics of these deaths have shifted over time. Drug toxicity was the single largest identified cause of death across all years, accounting for roughly 30 percent of fatalities in years where cause data was fully recorded, while men aged 40 to 59 represented the most affected demographic group. These findings point to the urgent need for targeted harm-reduction services and housing-first interventions for Toronto's most vulnerable residents.

1 Introduction

Homelessness in Toronto has grown into one of the city's most pressing public health challenges. As of recent counts, thousands of individuals rely on emergency shelters each night, and many more sleep outdoors or in encampments across the city. The consequences of life without stable housing are severe: people experiencing homelessness face exposure to extreme weather, barriers to health care, food insecurity, and elevated risks of violence and substance use. These cumulative stressors translate into dramatically shortened life expectancies relative to the housed population.

Despite growing public attention, the mortality burden of homelessness in Toronto has not been well quantified in accessible, data-driven terms. The City of Toronto began publicly tracking deaths of people experiencing homelessness in 2017 through its Open Data portal, providing

*Code and data are available at: https://github.com/EveHughes/Donaldson-Creator-and-Grader.

1

(a) Claude Opus 4.6, Project 5 (87%)

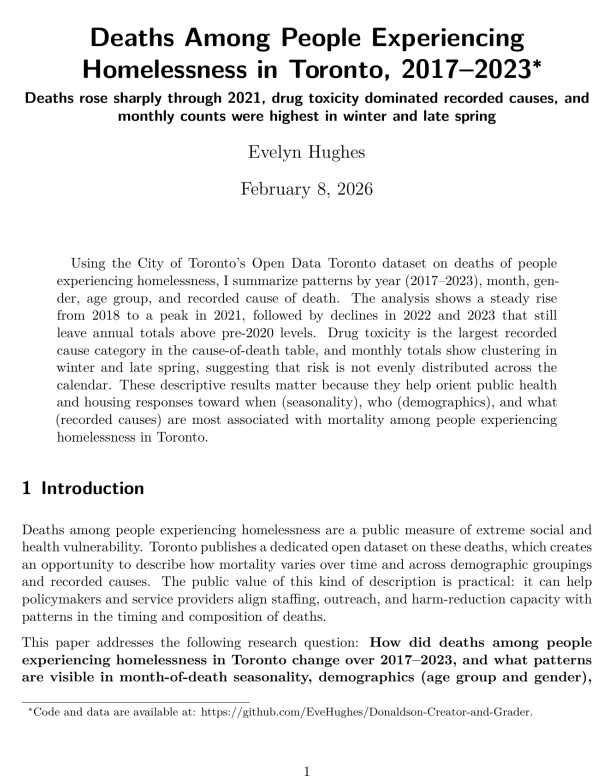

Deaths Among People Experiencing Homelessness in Toronto, 2017–2023*

Deaths rose sharply through 2021, drug toxicity dominated recorded causes, and monthly counts were highest in winter and late spring

Evelyn Hughes

February 8, 2026

Using the City of Toronto's Open Data Toronto dataset on deaths of people experiencing homelessness, I summarize patterns by year (2017–2023), month, gender, age group, and recorded cause of death. The analysis shows a steady rise from 2018 to a peak in 2021, followed by declines in 2022 and 2023 that still leave annual totals above pre-2020 levels. Drug toxicity is the largest recorded cause category in the cause-of-death table, and monthly totals show clustering in winter and late spring, suggesting that risk is not evenly distributed across the calendar. These descriptive results matter because they help orient public health and housing responses toward when (seasonality), who (demographics), and what (recorded causes) are most associated with mortality among people experiencing homelessness in Toronto.

1 Introduction

Deaths among people experiencing homelessness are a public measure of extreme social and health vulnerability. Toronto publishes a dedicated open dataset on these deaths, which creates an opportunity to describe how mortality varies over time and across demographic groupings and recorded causes. The public value of this kind of description is practical: it can help policymakers and service providers align staffing, outreach, and harm-reduction capacity with patterns in the timing and composition of deaths.

This paper addresses the following research question: **How did deaths among people experiencing homelessness in Toronto change over 2017–2023, and what patterns are visible in month-of-death seasonality, demographics (age group and gender),**

*Code and data are available at: https://github.com/EveHughes/Donaldson-Creator-and-Grader.

1

(b) GPT-5.2, Project 2 (85%)

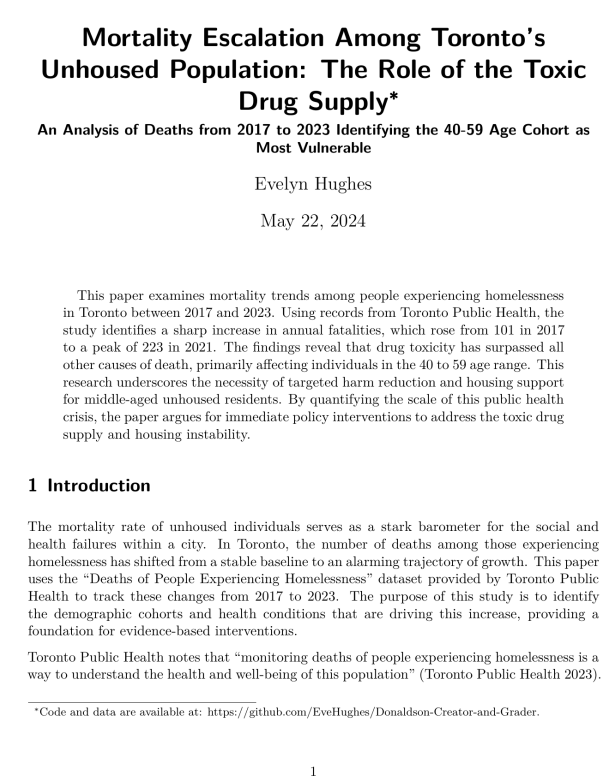

Mortality Escalation Among Toronto's Unhoused Population: The Role of the Toxic Drug Supply*

An Analysis of Deaths from 2017 to 2023 Identifying the 40-59 Age Cohort as Most Vulnerable

Evelyn Hughes

May 22, 2024

This paper examines mortality trends among people experiencing homelessness in Toronto between 2017 and 2023. Using records from Toronto Public Health, the study identifies a sharp increase in annual fatalities, which rose from 101 in 2017 to a peak of 223 in 2021. The findings reveal that drug toxicity has surpassed all other causes of death, primarily affecting individuals in the 40 to 59 age range. This research underscores the necessity of targeted harm reduction and housing support for middle-aged unhoused residents. By quantifying the scale of this public health crisis, the paper argues for immediate policy interventions to address the toxic drug supply and housing instability.

1 Introduction

The mortality rate of unhoused individuals serves as a stark barometer for the social and health failures within a city. In Toronto, the number of deaths among those experiencing homelessness has shifted from a stable baseline to an alarming trajectory of growth. This paper uses the "Deaths of People Experiencing Homelessness" dataset provided by Toronto Public Health to track these changes from 2017 to 2023. The purpose of this study is to identify the demographic cohorts and health conditions that are driving this increase, providing a foundation for evidence-based interventions.

Toronto Public Health notes that "monitoring deaths of people experiencing homelessness is a way to understand the health and well-being of this population" (Toronto Public Health 2023).

*Code and data are available at: https://github.com/EveHughes/Donaldson-Creator-and-Grader.

1

(c) Gemini 3 Flash, Project 3 (84%)

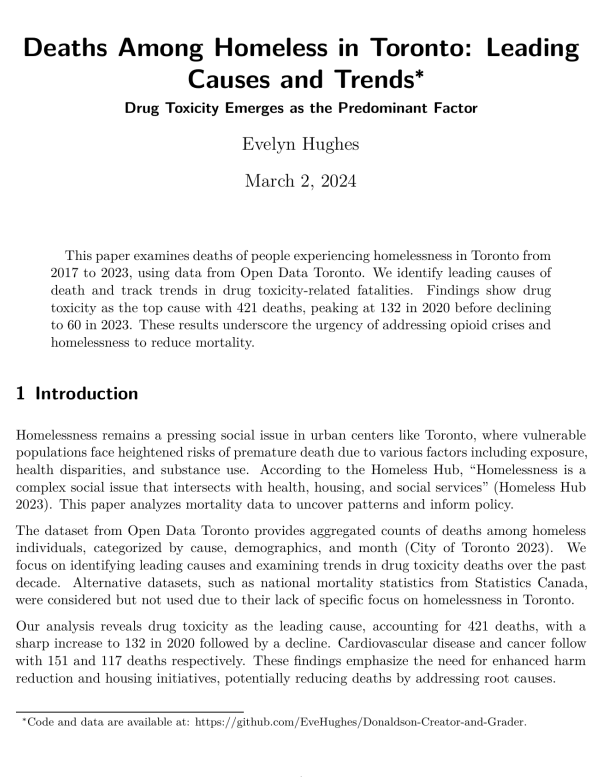

Deaths Among Homeless in Toronto: Leading Causes and Trends*

Drug Toxicity Emerges as the Predominant Factor

Evelyn Hughes

March 2, 2024

This paper examines deaths of people experiencing homelessness in Toronto from 2017 to 2023, using data from Open Data Toronto. We identify leading causes of death and track trends in drug toxicity-related fatalities. Findings show drug toxicity as the top cause with 421 deaths, peaking at 132 in 2020 before declining to 60 in 2023. These results underscore the urgency of addressing opioid crises and homelessness to reduce mortality.

1 Introduction

Homelessness remains a pressing social issue in urban centers like Toronto, where vulnerable populations face heightened risks of premature death due to various factors including exposure, health disparities, and substance use. According to the Homeless Hub, "Homelessness is a complex social issue that intersects with health, housing, and social services" (Homeless Hub 2023). This paper analyzes mortality data to uncover patterns and inform policy.

The dataset from Open Data Toronto provides aggregated counts of deaths among homeless individuals, categorized by cause, demographics, and month (City of Toronto 2023). We focus on identifying leading causes and examining trends in drug toxicity deaths over the past decade. Alternative datasets, such as national mortality statistics from Statistics Canada, were considered but not used due to their lack of specific focus on homelessness in Toronto.

Our analysis reveals drug toxicity as the leading cause, accounting for 421 deaths, with a sharp increase to 132 in 2020 followed by a decline. Cardiovascular disease and cancer follow with 151 and 117 deaths respectively. These findings emphasize the need for enhanced harm reduction and housing initiatives, potentially reducing deaths by addressing root causes.

*Code and data are available at: https://github.com/EveHughes/Donaldson-Creator-and-Grader.

1

(d) Grok-4, Project 5 (73%)

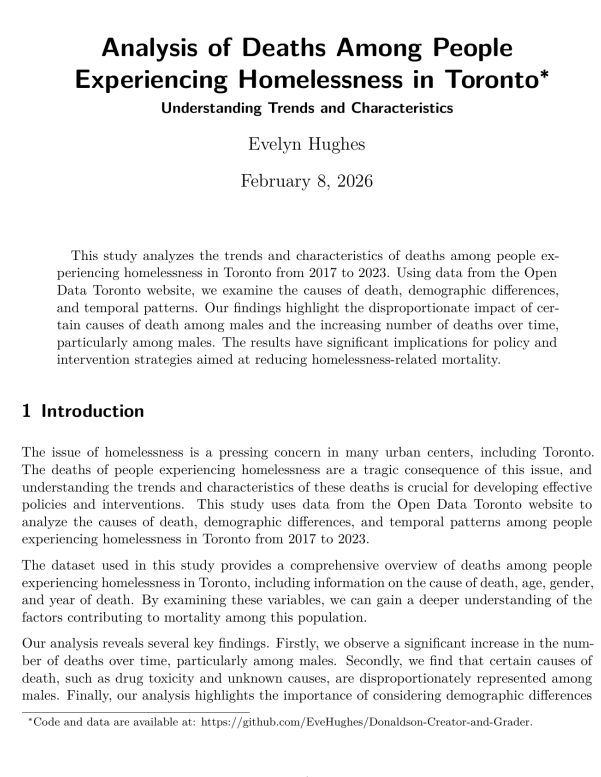

Analysis of Deaths Among People Experiencing Homelessness in Toronto*

Understanding Trends and Characteristics

Evelyn Hughes

February 8, 2026

This study analyzes the trends and characteristics of deaths among people experiencing homelessness in Toronto from 2017 to 2023. Using data from the Open Data Toronto website, we examine the causes of death, demographic differences, and temporal patterns. Our findings highlight the disproportionate impact of certain causes of death among males and the increasing number of deaths over time, particularly among males. The results have significant implications for policy and intervention strategies aimed at reducing homelessness-related mortality.

1 Introduction

The issue of homelessness is a pressing concern in many urban centers, including Toronto. The deaths of people experiencing homelessness are a tragic consequence of this issue, and understanding the trends and characteristics of these deaths is crucial for developing effective policies and interventions. This study uses data from the Open Data Toronto website to analyze the causes of death, demographic differences, and temporal patterns among people experiencing homelessness in Toronto from 2017 to 2023.

The dataset used in this study provides a comprehensive overview of deaths among people experiencing homelessness in Toronto, including information on the cause of death, age, gender, and year of death. By examining these variables, we can gain a deeper understanding of the factors contributing to mortality among this population.

Our analysis reveals several key findings. Firstly, we observe a significant increase in the number of deaths over time, particularly among males. Secondly, we find that certain causes of death, such as drug toxicity and unknown causes, are disproportionately represented among males. Finally, our analysis highlights the importance of considering demographic differences

*Code and data are available at: https://github.com/EveHughes/Donaldson-Creator-and-Grader.

1

(e) Llama 4, Project 5 (47%)

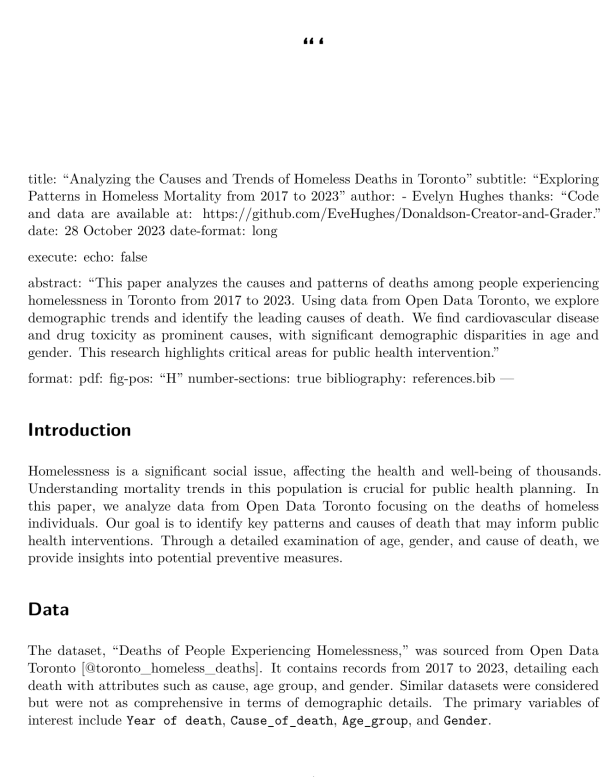

"'

title: "Analyzing the Causes and Trends of Homeless Deaths in Toronto" subtitle: "Exploring Patterns in Homeless Mortality from 2017 to 2023" author: - Evelyn Hughes thanks: "Code and data are available at: https://github.com/EveHughes/Donaldson-Creator-and-Grader." date: 28 October 2023 date-format: long

execute: echo: false

abstract: "This paper analyzes the causes and patterns of deaths among people experiencing homelessness in Toronto from 2017 to 2023. Using data from Open Data Toronto, we explore demographic trends and identify the leading causes of death. We find cardiovascular disease and drug toxicity as prominent causes, with significant demographic disparities in age and gender. This research highlights critical areas for public health intervention."

format: pdf: fig-pos: "H" number-sections: true bibliography: references.bib —

Introduction

Homelessness is a significant social issue, affecting the health and well-being of thousands. Understanding mortality trends in this population is crucial for public health planning. In this paper, we analyze data from Open Data Toronto focusing on the deaths of homeless individuals. Our goal is to identify key patterns and causes of death that may inform public health interventions. Through a detailed examination of age, gender, and cause of death, we provide insights into potential preventive measures.

Data

The dataset, "Deaths of People Experiencing Homelessness," was sourced from Open Data Toronto [@toronto_homeless_deaths]. It contains records from 2017 to 2023, detailing each death with attributes such as cause, age group, and gender. Similar datasets were considered but were not as comprehensive in terms of demographic details. The primary variables of interest include `Year of death`, `Cause_of_death`, `Age_group`, and `Gender`.

1

(f) GPT-4o, Project 2 (13%)

Fig 1. First pages of six generated papers.
