# Supplementary material for: Evaluating AI performance on end-to-end data science projects
Source: PLoS One. 2026 Nov 11;21(11):e0361887. doi: 10.1371/journal.pone.0361887 (PMC13754138; doi:10.1371/journal.pone.0361887)
Supplement: Supplementary file 4 [file pone.0361887.s004.pdf]

PONE-D-26-12071
Evaluating AI Performance on End-to-End Data Science Projects
PLOS One

Dear Dr. Alexander,

Thank you for submitting your manuscript to PLOS ONE. After careful consideration, we feel that it has merit but does not fully meet PLOS ONE's publication criteria as it currently stands. Therefore, we invite you to submit a revised version of the manuscript that addresses the points raised during the review process.

Please submit your revised manuscript by Jul 12 2026 11:59PM. If you will need more time than this to complete your revisions, please reply to this message or contact the journal office at plosone@plos.org. When you're ready to submit your revision, log on to https://www.editorialmanager.com/pone/ and select the 'Submissions Needing Revision' folder to locate your manuscript file.

Please include the following items when submitting your revised manuscript:

- A letter that responds to each point raised by the academic editor and reviewer(s). You should upload this letter as a separate file labeled 'Response to Reviewers'.
- A marked-up copy of your manuscript that highlights changes made to the original version. You should upload this as a separate file labeled 'Revised Manuscript with Track Changes'.
- An unmarked version of your revised paper without tracked changes. You should upload this as a separate file labeled 'Manuscript'.

If you would like to make changes to your financial disclosure, please include your updated statement in your cover letter. Guidelines for resubmitting your figure files are available below the reviewer comments at the end of this letter.

If applicable, we recommend that you deposit your laboratory protocols in protocols.io to enhance the reproducibility of your results. Protocols.io assigns your protocol its own identifier (DOI) so that it can be cited independently in the future. For instructions see: https://journals.plos.org/plosone/s/submission-guidelines#loc-laboratory-protocols. Additionally, PLOS ONE offers an option for publishing peer-reviewed Lab Protocol articles, which describe protocols hosted on protocols.io. Read more information on sharing protocols at https://plos.org/protocols?utm_medium=editorial-email&utm_source=authorletters&utm_campaign=protocols.

As the corresponding author, your ORCID iD is verified in the submission system and will appear in the published article. PLOS supports the use of ORCID, and we encourage all coauthors to register for an ORCID iD and use it as well. Please encourage your coauthors to verify their ORCID iD within the submission system before final acceptance, as unverified ORCID iDs will not appear in the published article. Only the individual author can complete the verification step; PLOS staff cannot verify ORCID iDs on behalf of authors.

We look forward to receiving your revised manuscript.

Kind regards,

Paolo Cazzaniga
Academic Editor
PLOS One

Journal Requirements:

When submitting your revision, we need you to address these additional requirements.
1. Please ensure that your manuscript meets PLOS ONE's style requirements, including those for file naming. The PLOS ONE style templates can be found at https://journals.plos.org/plosone/s/file?id=wjVg/PLOSOne_formatting_sample_main_body.pdf and https://journals.plos.org/plosone/s/file?id=ba62/PLOSOne_formatting_sample_title_authors_affiliations.pdf

Thank you for identifying these. We have attempted to go through and update everything (the main issue was the references and cross-references and line numbers and double spacing) but please let us know if we need to make more changes!

2. Please note that PLOS One has specific guidelines on code sharing for submissions in which author-generated code underpins the findings in the manuscript. In these cases, we expect all author-generated code to be made available without restrictions upon publication of the work. Please review our guidelines at https://journals.plos.org/plosone/s/materials-and-software-sharing#loc-sharing-code and ensure that your code is shared in a way that follows best practice and facilitates reproducibility and reuse.

We have uploaded all code here:
https://doi.org/10.17605/OSF.IO/WQSA7

3. When completing the data availability statement of the submission form, you indicated that you will make your data available on acceptance. We strongly recommend all authors decide on a data sharing plan before acceptance, as the process can be lengthy and hold up publication timelines. Please note that, though access restrictions are acceptable now, your entire data will need to be made freely accessible if your manuscript is accepted for publication. This policy applies to all data except where public deposition would breach compliance with the protocol approved by your research ethics board. If you are unable to adhere to our open data policy, please kindly revise your statement to explain your reasoning and we will seek the editor's input on an exemption. Please be assured that, once you have provided your new statement, the assessment of your exemption will not hold up the peer review process.

We thank you for this note. We have updated the statement and hope this is acceptable. We have made the underlying data available at that same OSF repo:
https://doi.org/10.17605/OSF.IO/WQSA7
Please note that we do not include the repos of the student work that our auto-grader is based on.

4. We note that the grant information you provided in the ‘Funding Information’ and ‘Financial Disclosure’ sections do not match.

When you resubmit, please ensure that you provide the correct grant numbers for the awards you received for your study in the ‘Funding Information’ section.

We apologize for this oversight and have corrected them.

5. Please include captions for your Supporting Information files at the end of your manuscript, and update any in-text citations to match accordingly. Please see our Supporting Information guidelines for more information: http://journals.plos.org/plosone/s/supporting-information.

Thank you for pointing this out. We have now included captions for Supporting Information files.

6. If the reviewer comments include a recommendation to cite specific previously published works, please review and evaluate these publications to determine whether they are relevant and should be cited. There is no requirement to cite these works unless the editor has indicated otherwise.

We have added the additional papers the reviewers suggested.

Additional Editor Comments :

The reviewers found the topic timely and relevant, and appreciated the use of authentic undergraduate data science assignments to evaluate LLM performance on realistic, open-ended tasks.
However, several issues should be addressed before the manuscript can be considered for publication.

A major concern relates to the validation of the LLM-as-a-judge framework. The current presentation does not sufficiently demonstrate the agreement between human and automatic grading, and reviewers recommend including clearer quantitative and graphical comparisons, as well as discussing the possibility of overfitting during prompt calibration.

We thank the editor for drawing our attention to this, and we have considerably re-written this section and hope that it is now more appropriate.

The presentation of the results should be strengthened overall. Reviewers suggest improving several figures (e.g., replacing bar plots with boxplots), moving some important appendix material into the main text, and providing a more explicit comparison between the different judge models, including possible judge-specific biases.

We thank the editor for highlighting these, and have followed the advice around figure improvements and moving content from appendices into main content.

The manuscript would also benefit from stronger positioning with respect to prior literature. In particular, the introduction should better contextualize related work on data science pipelines, benchmark design, and generative AI applications in data analytics. Additional methodological clarification is also needed regarding benchmark construction, project categorization, and rubric design.

We thank the editor for these directions and have improved this positioning.

Overall, the reviewers agree that the manuscript has potential and addresses an important problem, but substantial revisions are required to improve clarity, methodological transparency, and positioning of the contribution.

Thank you very much for your support and encouragement!

[Note: HTML markup is below. Please do not edit.]

Reviewers' comments:

Reviewer's Responses to Questions

Comments to the Author

1. Is the manuscript technically sound, and do the data support the conclusions?

The manuscript must describe a technically sound piece of scientific research with data that supports the conclusions. Experiments must have been conducted rigorously, with appropriate controls, replication, and sample sizes. The conclusions must be drawn appropriately based on the data presented.

Reviewer #1: Yes

Reviewer #2: Yes

2. Has the statistical analysis been performed appropriately and rigorously?

Reviewer #1: Yes

Reviewer #2: Yes

3. Have the authors made all data underlying the findings in their manuscript fully available?

The PLOS Data policy requires authors to make all data underlying the findings described in their manuscript fully available without restriction, with rare exception (please refer to the Data Availability Statement in the manuscript PDF file). The data should be provided as part of the manuscript or its supporting information, or deposited to a public repository. For example, in addition to summary statistics, the data points behind means, medians and variance measures should be available. If there are restrictions on publicly sharing data—e.g. participant privacy or use of data from a third party—those must be specified.

Reviewer #1: Yes

Reviewer #2: Yes

4. Is the manuscript presented in an intelligible fashion and written in standard English?

PLOS ONE does not copyedit accepted manuscripts, so the language in submitted articles must be clear, correct, and unambiguous. Any typographical or grammatical errors should be corrected at revision, so please note any specific errors here.

Reviewer #1: Yes

Reviewer #2: Yes

5. Review Comments to the Author

Please use the space provided to explain your answers to the questions above. You may also include additional comments for the author, including concerns about dual publication, research ethics, or publication ethics. (Please upload your review as an attachment if it exceeds 20,000 characters)

Reviewer #1: In their paper "Evaluating AI Performance on End-to-End Data Science Projects", the authors use various LLMs to generate data science projects, with task formulation identical to what students got in a undergraduate data science course. The authors also used (other) LLMs as a "judge" and adjusted the grading prompt until the automatic grades were close to the actual grades on completed students' projects. In the end they found that latest versions of Claude and ChatGPT on avarege achieve A-.

Strengths: Interesting and timely study. I appreciated the real tasks taken from real course assignments.

Weaknesses: (1) Most recent models are absent. (2) The LLM-as-a-judge is not fully validated. (3) Presentation of the results can be improved.

Overall, I recommend a revision. (3) can be easily fixed. Regarding (2) I am not sure if the authors have all the results already and just need to present them better, of if additional evaluations need to be run. For (1) the authors would need to run additional experiments; IMHO it is optional but would be nice to have.

MAJOR COMMENTS

* The latest models are Claude Opus 4.6 and GPT 5.2. But we have Opus 4.7 and GPT 5.5 for several months already. Can those be included into the revision?

We thank the reviewer for this suggestion. We added two models: Claude Opus 4.8 and GPT-5.4. All figures, tables, and the abstract now reflect nine models. We tried to include GPT-5.5, but it exhausted our pipeline's 100-attempt retry cap without producing a complete project. We have mentioned this in the paper.

* I can see some issues with autograding:

* The text contains sentence "The mean difference from the human-provided total mark for Claude Sonnet 4.5 was XXX points out of 45 and was XXX points out of 45 for GPT-5.2" -- please replace XXX with actual values!

We apologize for this embarrassing mistake and appreciate the reviewer identifying it. The values are now included.

* I feel you need to show these results more clearly, not just have one sentence about XXX points deviation. Maybe make a figure with all human vs automatic scores plotted as a scatter plot, one panel for each judge?

We thank the reviewer for this suggestion and have added a new main-text figure and results.

* I worry that there is no test-set evaluation... Prompts were adjusted on the training set (40 human projects), until the authors were satisfied with the result. There could have been some manual overfitting here. Having a test set (e.g. another 40 human projects) would be great, but I guess unrealistic (?). Not sure how to address this, but at least I would like this to be discussed.

This is a good point and we have added a paragraph into the Discussion. In general, we focused calibration on 10 of the 40 benchmark projects using GPT-4o, and then once that was finalized we tried GPT-5.2 and Sonnet 4.5 using those settings, and then picked the one that was best. We agree that a fully held-out human test set would be stronger and note this as a limitation.

* Figure 1 has very little content, please replace the barplot with a boxplot (you have 5 projects per model, so a boxplot could be based on these 5 points). Then you won't need Fig S1.

* Same can be done in Fig 2 and something similar can be done in Fig 3.

We thank the reviewer for this suggestion and have replaced Fig 1 and 2 with boxplots and removed Fig S1 as not needed.

* Why not show results from both judges directly in Figure 1? E.g. as two boxplots in two different colors next to each other? One would hope that the results agree between judges. Currently all consistency results are in the Appendix D. I think at least some of that should be moved into the main text.

We thank the reviewer for this suggestion and have moved the Appendix into main content. Only Sonnet 4.5 graded all the model-generated projects, and we now make that more explicit. We do have the two-judge comparison for the human-graded projects and also added some text about this.

* Also, you never show or say whether ChatGPT judge prefers ChatGPT projects. I think there should be full results for ChatGPT grading shown somewhere, and the difference in ranking between the two judges should be discussed.

We thank the reviewer for this suggestion. We agree this is an important concern and have expanded the Discussion. We used GPT-5.2 as a validation judge on the 40 human-written benchmark projects and they both basically agreed on the order although the marks from GPT-5.2 were lower. We also added into the paper that the scores for OpenAI were very similar to Anthropic's and that a different set-up would be important in future work.

MINOR COMMENTS

* Abstract: "Most recent models did well on structured tasks, but there were considerable differences on tasks that needed judgment" -- this is not enough detail. Please add some details here (what are "most recent models"? what does "did well" mean exactly? what "tasks that needed judgement"? what "differences"?). Abstract should convey the main findings.

We thank the reviewer for highlighting this. We have updated the abstract.

Reviewer #2: The authors present an interesting journal article in which they propose a new benchmark composed of data science projects, define evaluation rubric, and assess the performance of several LLMs in order to determine which models are better suited to evaluating real-world data science projects. A central challenge addressed by the paper is precisely the open-ended nature of these projects, since they can be approached through multiple valid methodological and technical paths. For this reason, the use of standardized starting points across projects, together with explicit rubric-based evaluation criteria, is a relevant strategy to make the assessment of LLM performance more consistent and comparable.

Overall, the article is promising and presents a valuable contribution. Nevertheless, I believe that several aspects require further attention and refinement before publication.

- The abstract is currently too short. It provides limited contextualization of the problem, and some passages are repeated almost identically in the introduction. I recommend expanding the abstract to better articulate the motivation, methodological contribution, main results, and implications of the study.

We thank the reviewer for pointing out that the abstract was insufficient. We have re-written it and hope that it is better.

- The introduction is generally well written, but in my view it does not sufficiently contextualize the broader problem being addressed. Since the manuscript does not include a specific State-of-the-Art or Related Work section, it becomes especially important to strengthen this discussion in the introduction. In particular, the authors should consider prior work that defines what a data science pipeline is, or should be, such as Biswas et al. (2022), “The art and practice of data science pipelines: A comprehensive study of data science pipelines in theory, in-the-small, and in-the-large.” In addition, it would be useful to discuss studies that explore the use of GenAI beyond programming tasks, for example in visualization and data analytics, such as Valverde-Rebaza et al. (2024), “Advanced large language models and visualization tools for data analytics learning.” More importantly, the manuscript should make its contribution with respect to the existing literature much more explicit.

We thank the reviewer for this comment. We have added mentions of both those papers and added an explicit paragraph about our contributions.

- The methodology is clear in general terms, but additional details are needed regarding the construction of the benchmark. It is not fully clear whether the benchmark design is entirely original or whether it was inspired by previous work. I recommend including a diagram that illustrates the complete benchmark construction process, from project selection and standardization to rubric design and LLM-based evaluation.

We thank the reviewer for this suggestion and have added the diagram, and noted this is original.

- Still regarding the benchmark construction, the rubric appears to include the production of a “paper” as one of its components. This point needs clarification. Should this be understood

as a technical report of the data science project? Not all data science projects naturally lead to the writing of an academic paper. I understand that, given the academic context in which the benchmark was developed, producing a paper may seem natural; however, this is not necessarily a standard component of a data science pipeline, particularly in industry-oriented settings.

This is a good point, and we thank the reviewer for noting this. We have added a paragraph about this into the Methods and Discussion sections.

- The methodology would also benefit from a clearer categorization of the projects included in the dataset. For example, are the projects classified as basic, intermediate, or advanced? Which stages of the data science lifecycle do they involve? Are they primarily academic projects, or do some of them reflect industry-oriented scenarios? This categorization is important to better interpret the performance of the LLMs evaluated in the study.

We thank the reviewer for this suggestion and have added a paragraph into the Methods section.

- It is also unclear whether the proposed rubric was adapted from existing rubrics in the literature, educational practice, or industry standards, or whether it was designed entirely by the authors. This should be explicitly stated. If the rubric is original, the authors should better justify its dimensions and criteria.

We now state the background of the rubric explicitly and map the different dimensions to aspects of the data science workflow.

- In the results section, the use of "XXX" appears in some passages, for example: "Claude Sonnet 4.5 was XXX points..." Is this a typo or placeholder text? This should be corrected before publication.

We apologize for this embarrassing mistake and thank the reviewer for noting it. We have fixed it.

- I would recommend moving several of the tables currently placed in the appendix into the main results section. These tables would enrich the discussion by providing direct evidence to the reader, without requiring constant consultation of the supplementary material.

We thank the reviewer for noting this, and have moved the details of the auto-grader and the different results for each model into the main paper.

- I suggest conducting an additional multivariate analysis of the results. This could help identify whether there is a more consolidated winning LLM, either considering the overall rubric score or specific rubric dimensions. Such analysis would strengthen the empirical claims of the paper and provide a more nuanced comparison across models.

We were a little concerned about adding a formal multivariate analysis because of the small n (both projects and models). But we have added discussion of the dimension

level rankings, which specifies that no single model wins all six dimensions. We hope this addresses the spirit of the suggestion.

- In the Discussion section, there is a citation written as “(Alexander 2023).” It seems that the author’s first name may have been used instead of the surname. Please verify and correct this reference if necessary.

We thank the reviewer for noting this. Alexander is a surname in this case.

- Finally, I recommend that the authors present the main findings more explicitly and position the novelty of the paper more clearly. The contribution is interesting, but the manuscript would benefit from a stronger closing argument explaining what is genuinely new, why it matters, and how this benchmark can support future research on LLM-based evaluation of data science projects.

We thank the reviewer for this suggestion. We have tried to update the introduction and discussion to make the contribution more clear.

Overall, the paper addresses a timely and relevant problem. With a stronger positioning in relation to prior work, more methodological transparency in the benchmark construction, and a deeper analysis of the results, the manuscript could make a solid contribution to the evaluation of LLMs in open-ended data science tasks.

We thank the reviewer for their encouragement and for taking the time to review our work.

6. PLOS authors have the option to publish the peer review history of their article (what does this mean?). If published, this will include your full peer review and any attached files.

If you choose “no”, your identity will remain anonymous but your review may still be made public.

Do you want your identity to be public for this peer review? For information about this choice, including consent withdrawal, please see our Privacy Policy.

Reviewer #1: No

Reviewer #2: Yes: JVR

[NOTE: If reviewer comments were submitted as an attachment file, they will be attached to this email and accessible via the submission site. Please log into your account, locate the manuscript record, and check for the action link "View Attachments". If this link does not appeatr, there are no attachment files.]

To ensure your figures meet our technical requirements, please review our figure guidelines: https://journals.plos.org/plosone/s/figures

You may also use PLOS's free figure tool, NAAS, to help you prepare publication quality figures: https://journals.plos.org/plosone/s/figures#loc-tools-for-figure-preparation.

NAAS will assess whether your figures meet our technical requirements by comparing each figure against our figure specifications.

In compliance with data protection regulations, you may request that we remove your personal registration details at any time. (Remove my information/details). Please contact the publication office if you have any questions.
