# Supplementary material for: Evaluating AI performance on end-to-end data science projects
Source: PLoS One. 2026 Nov 11;21(11):e0361887. doi: 10.1371/journal.pone.0361887 (PMC13754138; doi:10.1371/journal.pone.0361887)
Supplement: Peer review history

# Peer review: Decision Letter 0

**DOI:** 10.1371/journal.pone.0361887.r001

**Authors:** Paolo Cazzaniga

28 May 2026

Dear Dr. Alexander,

Please submit your revised manuscript by Jul 12 2026 11:59PM. If you will need more time than this to complete your revisions, please reply to this message or contact the journal office at plosone@plos.org. When you're ready to submit your revision, log on to https://www.editorialmanager.com/pone/ and select the 'Submissions Needing Revision' folder to locate your manuscript file.

- A letter that responds to each point raised by the academic editor and reviewer(s). You should upload this letter as a separate file labeled 'Response to Reviewers'.
- A marked-up copy of your manuscript that highlights changes made to the original version. You should upload this as a separate file labeled 'Revised Manuscript with Track Changes'.
- An unmarked version of your revised paper without tracked changes. You should upload this as a separate file labeled 'Manuscript'.

If you would like to make changes to your financial disclosure, please include your updated statement in your cover letter. Guidelines for resubmitting your figure files are available below the reviewer comments at the end of this letter.

If applicable, we recommend that you deposit your laboratory protocols in protocols.io to enhance the reproducibility of your results. Protocols.io assigns your protocol its own identifier (DOI) so that it can be cited independently in the future. For instructions see: https://journals.plos.org/plosone/s/submission-guidelines#loc-laboratory-protocols. Additionally, PLOS ONE offers an option for publishing peer-reviewed Lab Protocol articles, which describe protocols hosted on protocols.io. Read more information on sharing protocols at https://plos.org/protocols?utm_medium=editorial-email&utm_source=authorletters&utm_campaign=protocols.

As the corresponding author, your ORCID iD is verified in the submission system and will appear in the published article. PLOS supports the use of ORCID, and we encourage all coauthors to register for an ORCID iD and use it as well. Please encourage your coauthors to verify their ORCID iD within the submission system before final acceptance, as unverified ORCID iDs will not appear in the published article. *Only*  the individual author can complete the verification step; PLOS staff *cannot*  verify ORCID iDs on behalf of authors.

We look forward to receiving your revised manuscript.

Kind regards,

Paolo Cazzaniga

Academic Editor

PLOS One

Journal Requirements:

When submitting your revision, we need you to address these additional requirements.

1. Please ensure that your manuscript meets PLOS ONE's style requirements, including those for file naming. The PLOS ONE style templates can be found at

https://journals.plos.org/plosone/s/file?id=wjVg/PLOSOne_formatting_sample_main_body.pdf and https://journals.plos.org/plosone/s/file?id=ba62/PLOSOne_formatting_sample_title_authors_affiliations.pdf

2. Please note that PLOS One has specific guidelines on code sharing for submissions in which author-generated code underpins the findings in the manuscript. In these cases, we expect all author-generated code to be made available without restrictions upon publication of the work. Please review our guidelines at https://journals.plos.org/plosone/s/materials-and-software-sharing#loc-sharing-code and ensure that your code is shared in a way that follows best practice and facilitates reproducibility and reuse.

3. When completing the data availability statement of the submission form, you indicated that you will make your data available on acceptance. We strongly recommend all authors decide on a data sharing plan before acceptance, as the process can be lengthy and hold up publication timelines. Please note that, though access restrictions are acceptable now, your entire data will need to be made freely accessible if your manuscript is accepted for publication. This policy applies to all data except where public deposition would breach compliance with the protocol approved by your research ethics board. If you are unable to adhere to our open data policy, please kindly revise your statement to explain your reasoning and we will seek the editor's input on an exemption. Please be assured that, once you have provided your new statement, the assessment of your exemption will not hold up the peer review process.

4. We note that the grant information you provided in the ‘Funding Information’ and ‘Financial Disclosure’ sections do not match.

When you resubmit, please ensure that you provide the correct grant numbers for the awards you received for your study in the ‘Funding Information’ section.

5. Please include captions for your Supporting Information files at the end of your manuscript, and update any in-text citations to match accordingly. Please see our Supporting Information guidelines for more information: http://journals.plos.org/plosone/s/supporting-information.

6. If the reviewer comments include a recommendation to cite specific previously published works, please review and evaluate these publications to determine whether they are relevant and should be cited. There is no requirement to cite these works unless the editor has indicated otherwise.

Additional Editor Comments :

The reviewers found the topic timely and relevant, and appreciated the use of authentic undergraduate data science assignments to evaluate LLM performance on realistic, open-ended tasks.

However, several issues should be addressed before the manuscript can be considered for publication.

A major concern relates to the validation of the LLM-as-a-judge framework. The current presentation does not sufficiently demonstrate the agreement between human and automatic grading, and reviewers recommend including clearer quantitative and graphical comparisons, as well as discussing the possibility of overfitting during prompt calibration.

The presentation of the results should be strengthened overall. Reviewers suggest improving several figures (e.g., replacing bar plots with boxplots), moving some important appendix material into the main text, and providing a more explicit comparison between the different judge models, including possible judge-specific biases.

The manuscript would also benefit from stronger positioning with respect to prior literature. In particular, the introduction should better contextualize related work on data science pipelines, benchmark design, and generative AI applications in data analytics. Additional methodological clarification is also needed regarding benchmark construction, project categorization, and rubric design.

Overall, the reviewers agree that the manuscript has potential and addresses an important problem, but substantial revisions are required to improve clarity, methodological transparency, and positioning of the contribution.

[Note: HTML markup is below. Please do not edit.]

Reviewers' comments:

Reviewer's Responses to Questions

**Comments to the Author**

1. Is the manuscript technically sound, and do the data support the conclusions?

Reviewer #1: Yes

Reviewer #2: Yes

**********

2. Has the statistical analysis been performed appropriately and rigorously?

Reviewer #1: Yes

Reviewer #2: Yes

**********

3. Have the authors made all data underlying the findings in their manuscript fully available?

The PLOS Data policy

Reviewer #1: Yes

Reviewer #2: Yes

**********

4. Is the manuscript presented in an intelligible fashion and written in standard English?

Reviewer #1: Yes

Reviewer #2: Yes

**********

Reviewer #1: In their paper "Evaluating AI Performance on End-to-End Data Science Projects", the authors use various LLMs to generate data science projects, with task formulation identical to what students got in a undergraduate data science course. The authors also used (other) LLMs as a "judge" and adjusted the grading prompt until the automatic grades were close to the actual grades on completed students' projects. In the end they found that latest versions of Claude and ChatGPT on avarege achieve A-.

Strengths: Interesting and timely study. I appreciated the real tasks taken from real course assignments.

Weaknesses: (1) Most recent models are absent. (2) The LLM-as-a-judge is not fully validated. (3) Presentation of the results can be improved.

Overall, I recommend a revision. (3) can be easily fixed. Regarding (2) I am not sure if the authors have all the results already and just need to present them better, of if additional evaluations need to be run. For (1) the authors would need to run additional experiments; IMHO it is optional but would be nice to have.

MAJOR COMMENTS

* The latest models are Claude Opus 4.6 and GPT 5.2. But we have Opus 4.7 and GPT 5.5 for several months already. Can those be included into the revision?

* I can see some issues with autograding:

* The text contains sentence "The mean difference from the human-provided total mark for Claude Sonnet 4.5 was XXX points out of 45 and was XXX points out of 45 for GPT-5.2" -- please replace XXX with actual values!

* I feel you need to show these results more clearly, not just have one sentence about XXX points deviation. Maybe make a figure with all human vs automatic scores plotted as a scatter plot, one panel for each judge?

* I worry that there is no test-set evaluation... Prompts were adjusted on the training set (40 human projects), until the authors were satisfied with the result. There could have been some manual overfitting here. Having a test set (e.g. another 40 human projects) would be great, but I guess unrealistic (?). Not sure how to address this, but at least I would like this to be discussed.

* Figure 1 has very little content, please replace the barplot with a boxplot (you have 5 projects per model, so a boxplot could be based on these 5 points). Then you won't need Fig S1.

* Same can be done in Fig 2 and something similar can be done in Fig 3.

* Why not show results from both judges directly in Figure 1? E.g. as two boxplots in two different colors next to each other? One would hope that the results agree between judges. Currently all consistency results are in the Appendix D. I think at least some of that should be moved into the main text.

* Also, you never show or say whether ChatGPT judge prefers ChatGPT projects. I think there should be full results for ChatGPT grading shown somewhere, and the difference in ranking between the two judges should be discussed.

MINOR COMMENTS

* Abstract: "Most recent models did well on structured tasks, but there were considerable differences on tasks that needed judgment" -- this is not enough detail. Please add some details here (what are "most recent models"? what does "did well" mean exactly? what "tasks that needed judgement"? what "differences"?). Abstract should convey the main findings.

Reviewer #2: The authors present an interesting journal article in which they propose a new benchmark composed of data science projects, define evaluation rubric, and assess the performance of several LLMs in order to determine which models are better suited to evaluating real-world data science projects. A central challenge addressed by the paper is precisely the open-ended nature of these projects, since they can be approached through multiple valid methodological and technical paths. For this reason, the use of standardized starting points across projects, together with explicit rubric-based evaluation criteria, is a relevant strategy to make the assessment of LLM performance more consistent and comparable.

Overall, the article is promising and presents a valuable contribution. Nevertheless, I believe that several aspects require further attention and refinement before publication.

- The abstract is currently too short. It provides limited contextualization of the problem, and some passages are repeated almost identically in the introduction. I recommend expanding the abstract to better articulate the motivation, methodological contribution, main results, and implications of the study.

- The introduction is generally well written, but in my view it does not sufficiently contextualize the broader problem being addressed. Since the manuscript does not include a specific State-of-the-Art or Related Work section, it becomes especially important to strengthen this discussion in the introduction. In particular, the authors should consider prior work that defines what a data science pipeline is, or should be, such as Biswas et al. (2022), “The art and practice of data science pipelines: A comprehensive study of data science pipelines in theory, in-the-small, and in-the-large.” In addition, it would be useful to discuss studies that explore the use of GenAI beyond programming tasks, for example in visualization and data analytics, such as Valverde-Rebaza et al. (2024), “Advanced large language models and visualization tools for data analytics learning.” More importantly, the manuscript should make its contribution with respect to the existing literature much more explicit.

- The methodology is clear in general terms, but additional details are needed regarding the construction of the benchmark. It is not fully clear whether the benchmark design is entirely original or whether it was inspired by previous work. I recommend including a diagram that illustrates the complete benchmark construction process, from project selection and standardization to rubric design and LLM-based evaluation.

- Still regarding the benchmark construction, the rubric appears to include the production of a “paper” as one of its components. This point needs clarification. Should this be understood as a technical report of the data science project? Not all data science projects naturally lead to the writing of an academic paper. I understand that, given the academic context in which the benchmark was developed, producing a paper may seem natural; however, this is not necessarily a standard component of a data science pipeline, particularly in industry-oriented settings.

- The methodology would also benefit from a clearer categorization of the projects included in the dataset. For example, are the projects classified as basic, intermediate, or advanced? Which stages of the data science lifecycle do they involve? Are they primarily academic projects, or do some of them reflect industry-oriented scenarios? This categorization is important to better interpret the performance of the LLMs evaluated in the study.

- It is also unclear whether the proposed rubric was adapted from existing rubrics in the literature, educational practice, or industry standards, or whether it was designed entirely by the authors. This should be explicitly stated. If the rubric is original, the authors should better justify its dimensions and criteria.

- In the results section, the use of “XXX” appears in some passages, for example: “Claude Sonnet 4.5 was XXX points...” Is this a typo or placeholder text? This should be corrected before publication.

- I would recommend moving several of the tables currently placed in the appendix into the main results section. These tables would enrich the discussion by providing direct evidence to the reader, without requiring constant consultation of the supplementary material.

- I suggest conducting an additional multivariate analysis of the results. This could help identify whether there is a more consolidated winning LLM, either considering the overall rubric score or specific rubric dimensions. Such analysis would strengthen the empirical claims of the paper and provide a more nuanced comparison across models.

- In the Discussion section, there is a citation written as “(Alexander 2023).” It seems that the author’s first name may have been used instead of the surname. Please verify and correct this reference if necessary.

- Finally, I recommend that the authors present the main findings more explicitly and position the novelty of the paper more clearly. The contribution is interesting, but the manuscript would benefit from a stronger closing argument explaining what is genuinely new, why it matters, and how this benchmark can support future research on LLM-based evaluation of data science projects.

Overall, the paper addresses a timely and relevant problem. With a stronger positioning in relation to prior work, more methodological transparency in the benchmark construction, and a deeper analysis of the results, the manuscript could make a solid contribution to the evaluation of LLMs in open-ended data science tasks.

**********

what does this mean?). If published, this will include your full peer review and any attached files.

If you choose “no”, your identity will remain anonymous but your review may still be made public.

**Do you want your identity to be public for this peer review?**  For information about this choice, including consent withdrawal, please see our Privacy Policy

Reviewer #1: No

Reviewer #2: **Yes:**  JVR

**********

[NOTE: If reviewer comments were submitted as an attachment file, they will be attached to this email and accessible via the submission site. Please log into your account, locate the manuscript record, and check for the action link "View Attachments". If this link does not appear, there are no attachment files.]

To ensure your figures meet our technical requirements, please review our figure guidelines: https://journals.plos.org/plosone/s/figures

You may also use PLOS’s free figure tool, NAAS, to help you prepare publication quality figures: https://journals.plos.org/plosone/s/figures#loc-tools-for-figure-preparation.

NAAS will assess whether your figures meet our technical requirements by comparing each figure against our figure specifications.

# Author comment: Author response to Decision Letter 1

**DOI:** 10.1371/journal.pone.0361887.r002

14 Aug 2026

We hope it is okay that we included our responses in the reviewer response PDF.

Attachment Submitted filename: 2026-07-12-response.pdf

# Peer review: Decision Letter 1

**DOI:** 10.1371/journal.pone.0361887.r003

**Authors:** Carla Pegoraro

19 Oct 2026

Evaluating AI performance on end-to-end data science projects

PONE-D-26-12071R1

Dear Dr. Alexander,

We’re pleased to inform you that your manuscript has been judged scientifically suitable for publication and will be formally accepted for publication once it meets all outstanding technical requirements.

Within one week, you’ll receive an e-mail detailing the required amendments. When these have been addressed, you’ll receive a formal acceptance letter and your manuscript will be scheduled for publication.

An invoice will be generated when your article is formally accepted. Please note, if your institution has a publishing partnership with PLOS and your article meets the relevant criteria, all or part of your publication costs will be covered. Please make sure your user information is up-to-date by logging into Editorial Manager at Editorial Manager® and clicking the ‘Update My Information' link at the top of the page. For questions related to billing, please contact billing support.

If your institution or institutions have a press office, please notify them about your upcoming paper to help maximize its impact. If they’ll be preparing press materials, please inform our press team as soon as possible -- no later than 48 hours after receiving the formal acceptance. Your manuscript will remain under strict press embargo until 2 pm Eastern Time on the date of publication. For more information, please contact onepress@plos.org.

Kind regards,

Carla Pegoraro

Staff Editor

PLOS One

Additional Editor Comments (optional):

Reviewers' comments:

Reviewer's Responses to Questions

**Comments to the Author**

Reviewer #1: All comments have been addressed

**********

2. Is the manuscript technically sound, and do the data support the conclusions?

Reviewer #1: Yes

**********

3. Has the statistical analysis been performed appropriately and rigorously?

Reviewer #1: Yes

**********

4. Have the authors made all data underlying the findings in their manuscript fully available?

The PLOS Data policy

Reviewer #1: Yes

**********

5. Is the manuscript presented in an intelligible fashion and written in standard English?

Reviewer #1: Yes

**********

Reviewer #1: I thank the authors for addressing my comments. I think the manuscript can be published as is. I would recommend the authors to remove ", yet OpenAI's GPT-5.5 was unable to complete the task" from the abstract, because it attracts a lot of attention to this statement, while it seems to me that it can be dependent on the exact prompt and can potentially be fixed. In the main manuscript I would suggest to have some more details about why "retries" were needed at all and in what sense GPT 5.5 was unable to complete the task (runtime timeout? or what happened exactly?).

**********

what does this mean?). If published, this will include your full peer review and any attached files.

If you choose “no”, your identity will remain anonymous but your review may still be made public.

**Do you want your identity to be public for this peer review?**  For information about this choice, including consent withdrawal, please see our Privacy Policy

Reviewer #1: No

**********

# Editor report: Acceptance letter

**DOI:** 10.1371/journal.pone.0361887.r004

**Authors:** Carla Pegoraro

PONE-D-26-12071R1

PLOS One

Dear Dr. Alexander,

I'm pleased to inform you that your manuscript has been deemed suitable for publication in PLOS One. Congratulations! Your manuscript is now being handed over to our production team.

At this stage, our production department will prepare your paper for publication. This includes ensuring the following:

* All references, tables, and figures are properly cited

* All relevant supporting information is included in the manuscript submission,

* There are no issues that prevent the paper from being properly typeset

You will receive further instructions from the production team, including instructions on how to review your proof when it is ready. Please keep in mind that we are working through a large volume of accepted articles, so please give us a few days to review your paper and let you know the next and final steps.

Lastly, if your institution or institutions have a press office, please let them know about your upcoming paper now to help maximize its impact. If they'll be preparing press materials, please inform our press team within the next 48 hours. Your manuscript will remain under strict press embargo until 2 pm Eastern Time on the date of publication. For more information, please contact onepress@plos.org.

You will receive an invoice from PLOS for your publication fee after your manuscript has reached the completed accept phase. If you receive an email requesting payment before acceptance or for any other service, this may be a phishing scheme. Learn how to identify phishing emails and protect your accounts at https://explore.plos.org/phishing.

If we can help with anything else, please email us at customercare@plos.org.

Thank you for submitting your work to PLOS One and supporting open access.

Kind regards,

PLOS One Editorial Office Staff

on behalf of

Dr Carla Pegoraro

Staff Editor

PLOS One
